# Automated CT dataset generation as a novel concept for verification of backshape-to-spine approach and Cobb Angle Estimation

**DOI:** 10.1371/journal.pone.0353213

**Published:** 2026-07-17

**Authors:** David Fräulin, Irina Sidorenko, Renée Lampe

**Affiliations:** 1 Department of Clinical Medicine, Center for Digital Health and Technology, Orthopedic Department, Research Unit for Paediatric Neuroorthopedics and Cerebral Palsy of the Buhl-Strohmaier Foundation, Klinikum rechts der Isar, School of Medicine and Health, Technical University of Munich, Munich, Germany; 2 Department of Clinical Medicine, Center for Digital Health and Technology, Orthopedic Department, Research Unit for Paediatric Neuroorthopedics and Cerebral Palsy of the Buhl-Strohmaier Foundation, Klinikum rechts der Isar, School of Medicine and Health, Technical University of Munich, Munich, Germany; 3 Department of Clinical Medicine, Center for Digital Health and Technology, Orthopedic Department, Research Unit for Paediatric Neuroorthopedics and Cerebral Palsy of the Buhl-Strohmaier Foundation, Markus Würth Professorship, Klinikum rechts der Isar, School of Medicine and Health, Technical University of Munich, Munich, Germany; Politecnico di Torino, ITALY

## Abstract

**Background:**

Adolescent idiopathic scoliosis is a condition that affects up to 3% of adolescents. Although radiographic imaging is the gold standard for diagnosis and monitoring, repeated exposure to ionizing radiation poses significant health risks. Alternative methods, such as estimating spinal alignment and Cobb angle from back surface topography, have been proposed to mitigate these risks. However, validation of these methods is challenging because traditional datasets combine back surface scans and radiographs acquired at different times and in different postures. This introduces technique-dependent errors and makes it difficult to determine whether observed discrepancies stem from the method itself or from inconsistencies in the validation dataset. As a result, this uncertainty in methods accuracy arising from verification errors, limits these alternatives to complementary roles rather than establishing them as reliable diagnostic tools.

**Methods:**

To address this limitation, we introduce a fully automated approach for generating verification datasets directly from computed tomography images. This approach enables precise and synchronized extraction of both external back surface and internal spinal structures, thereby eliminating posture-related discrepancies present in commonly used datasets. We assess the utility of our method through a two-step validation: first, by comparing automatically extracted parameters with manual measurements; and second, by comparing the results of an established surface topography method applied to our dataset with reported values from the literature.

**Results:**

Our findings suggest that this automatically extracted CT dataset serves as a suitable reference standard for the verification and comparison of surface topography approaches.

**Conclusion:**

This work provides a robust framework for future verification studies of different surface topography methods and may facilitate the development of non-radiographic techniques for scoliosis assessment, thereby reducing reliance on ionizing radiation in clinical practice.

## 1 Introduction

Adolescent Idiopathic Scoliosis (AIS) affects up to 3% of the population aged 10–18 years, with a notably higher prevalence among females (2–4%) [1]. The condition demands systematic diagnostic and monitoring approaches, with radiographic examination serving as the current gold standard. This examination primarily determines the coronal internal spinal alignment (ISL) and Cobb angle [2], which remain fundamental metrics for treatment decisions and progression monitoring [3].

However, the cumulative radiation exposure from repeated radiographs presents significant health concerns, particularly for young female patients who show increased susceptibility to radiation-induced cancer [4,5]. A systematic review and meta-analysis found that patients with scoliosis underwent an average of 23 full-spine radiographs during the observation period. Compared to the general population, these patients had a 46% higher risk of developing cancer, and women with scoliosis had a 20% increased risk of breast cancer [5]. These risks have motivated the development of various non-radiographic approaches, with Surface Topography (ST) becoming a widely used method [6–9], due to the development of inexpensive, accurate, and handheld 3D scanners [10–13].

ST methods have been developed based on the assumption that ST accurately represents the underlying spinal deformity, as scoliosis is a three-dimensional deformation of the spine that induces geometric and morphologic changes in the trunk and rib cage [14]. Each of the ST methods consist of two primary components: the technology for raw data acquisition (RDA) and the surface topography analysis method (STAM). Since RDA utilizes general-purpose 3D scanning technologies that are established across various industries and have demonstrated high accuracy in capturing external body contours [15,16], this work focuses exclusively on the STAM used for deriving meaningful disease describing parameters.

Research on STAM has evolved along different paths aiming to solve three distinct problems: early detection in large populations [17–22], progression monitoring during long-term follow-up to indicate when a radiographic examination is necessary [23,24], and potential replacement of radiographic methods through prediction of radiographic equivalent parameters with clinically acceptable accuracy. STAM addressing this final objective are further subdivided into two categories: direct prediction of the Cobb angle [8,25–29] and prediction of the ISL from which the Cobb angle can be derived. The latter is herein referred to as the *backshape-to-spine approach* [6,9,30–32].

Notably, studies investigating this approach have reported contradictory results. On one hand, diverse analytical approaches suggest that surface data contains sufficient information to predict Cobb angles with potentially high accuracy, as demonstrated by mean absolute error (MAE) and standard deviation (SD) values reported in the literature: (MAE = 6.2°) [8], (MAE = 3.177°, SD = 1.752°) [26], (MAE = 4.0°, SD = 5.4°) [33], (SD = 7.9°) [6], and (MAE = 3.42°, SD = 2.64°) [32]. These results indicate accuracy levels that may exceed the intra-observer MAE margins of traditional radiographic methods (2°–15.1° [34–36]). On the other hand, validation studies comparing Cobb angles derived from DIERS Formetric 4D system (DIERS International GmbH, Schlangenbad, Germany) with radiographic measurements from EOS imaging (EOS Imaging SA, Paris, France) reported only moderate correlations [37–39]. This discrepancy between surface-based estimations and actual internal spine measurements currently limits ST approaches to serving as complementary tools rather than definitive diagnostic alternatives to radiography [40].

The validity of STAM predictions depends critically on the alignment between back surface scans and radiographic references within validation datasets. If the data contains posture-related discrepancies, these errors become embedded in the STAM. Most studies [6,31,32,37–39,41] failed to ensure identical postures while obtaining the back surface scans and radiographic references, introducing technique-dependent errors. Furthermore, conventional 2D radiographs are limited in the anatomical measurements they can provide, because most 3D measurements, such as axial vertebral rotation, can not be accurately measured from 2D radiographs [42]. Although recent protocols using the EOS system [9] mitigate 2D projection errors, reliance on manual positioning and temporal gaps between acquisitions means that residual alignment errors still persist.

To the best of our knowledge, no study has yet utilized datasets that fully overcome these alignment errors and allow for the accurate determination of all necessary reference parameters. To address this gap, we present a novel approach for deriving datasets for the validation of any backshape-to-spine approach using computed tomography (CT) imaging. Leveraging the high accuracy of CT—capturing vertebral dimensions within 1 mm [43], centroids within 1.8 mm [44], and rotation within 1.0° [45]— our method offers two fundamental advantages. First, it enables retrospective collection from standard medical scans with complete synchronization between external back surface and internal spinal structures, effectively eliminating posture-related discrepancies. Second, it extracts comprehensive anatomical parameters—including axial rotation—providing a level of validation that was previously unattainable with radiographic datasets. To demonstrate the utility of this approach, we perform a two-step validation: first, by comparing our automatically extracted parameters with expert manual measurements; and second, by benchmarking the error profiles of an established STAM, Drerup’s backshape-to-spine method [6] (commercially implemented in the DIERS Formetric 4D system) applied to our CT-extracted dataset against values reported in the literature.

## 2 Materials and methods

This section describes the CT data used to develop the extraction methodology for a backshape-to-spine approach dataset, comprising both the patient’s skin surface and measurements of corresponding internal spinal structures. It then details the fully automated process for extracting these required data elements from CT scans. Finally, the section demonstrates the application of our Drerup-based implementation to the extracted dataset.

### 2.1 CT-data

For this study, 17 axial CT scans of the thorax and abdomen were analyzed from a mixed-gender patient cohort (Patients 1–17, P1–P17). Patient ages ranged from 54 to 88 years. The CT data were retrospectively selected from existing images at the radiology department, where scans were originally performed for other medical purposes but incidentally showed spinal deformations, as confirmed by a senior orthopedic specialist. The data was accessed from 2017 until 2025 and was collected by the radiology department. The sole inclusion criterion was a visually lean patient habitus, resulting in a random distribution of deformity types and severities. They were anonymized prior to transfer to the research team, ensuring that the authors had no access to information that could identify the patient during or after the data collection. No additional CT scans were performed for this study.

To provide a comprehensive cohort description, each scan was classified by the same senior orthopedic specialist according to two criteria: curvature severity and affected spinal region. The severity classification used four categories based on Cobb angle measurements: spinal curve (<10°), mild (10–20°), moderate (20–40°), and severe (>40°) [46]. The affected spinal regions were categorized as: (a) high thoracic (apex between ${\text{T}}_{2}-{\text{T}}_{5}$), (b) thoracic (apex between ${\text{T}}_{6}-{\text{T}}_{11}$), (c) thoraco-lumbar (apex around ${\text{T}}_{12}-{\text{L}}_{1}$), (d) lumbar (apex between ${\text{L}}_{2}-{\text{L}}_{5}$). The detailed distribution of patients across these categories is provided in Table A1 in S1 Supplementary Information. However, due to the limited sample size of 17 patients resulting in single-subject representation in certain subgroups, the cohort was analyzed as a single entity.

All CT data were anonymized by the radiology department, and the study protocol was approved by the ethics committee of the Faculty of Medicine of the Technical University of Munich (Eth.nr. 569/16 S).

### 2.2 Independent implementation of the backshape-to-spine approach based on the principles described by Drerup

**Fig 1:**
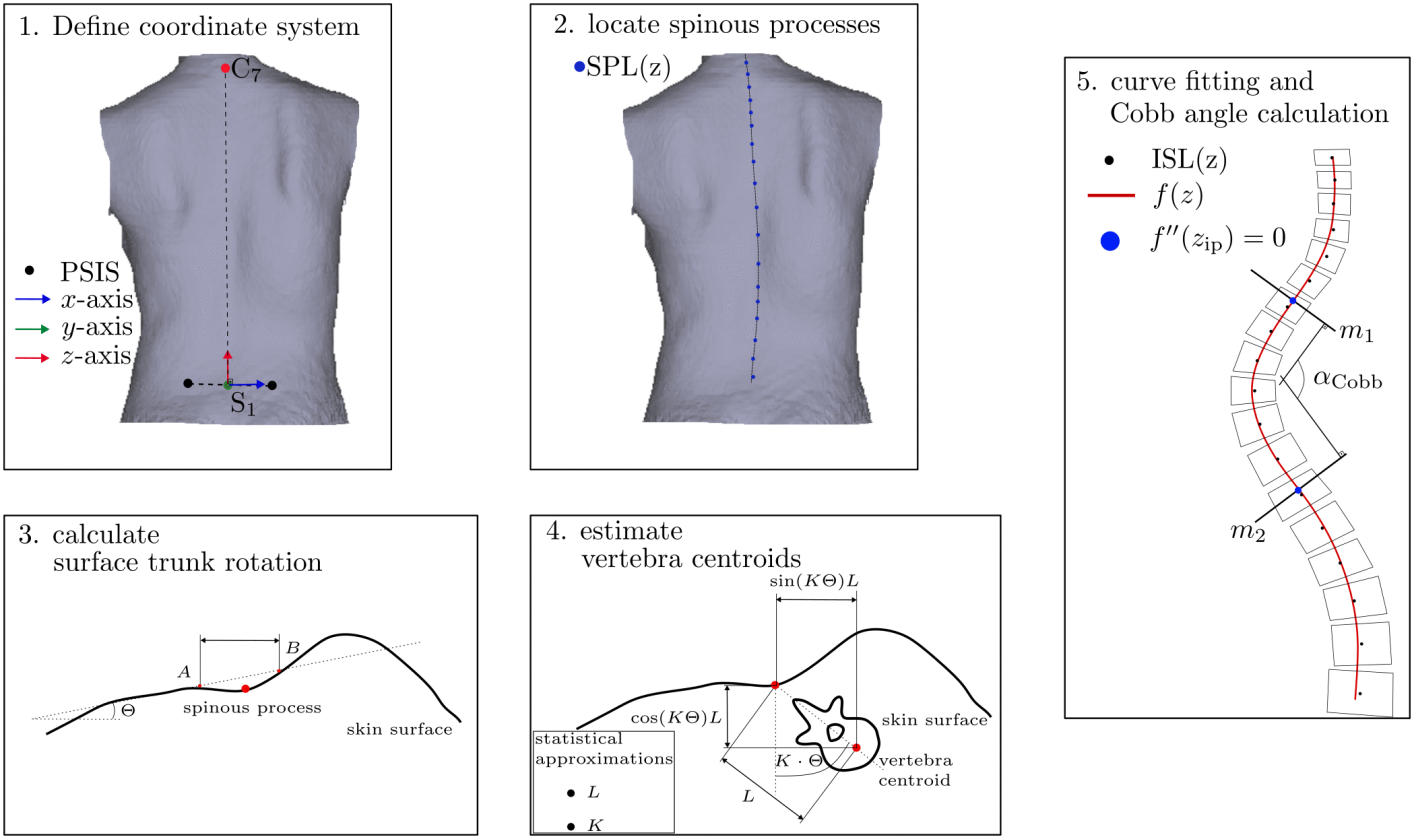
Step by step overview of Drerup’s backshape-to-spine approach. (1) Reference coordinate system definition. (2) Estimation of the spinous process line (SPL). (3) Calculation of the surface trunk rotation (STR) angle. (4) Estimation of distance *L* and factor *K*. (5) Calculation of the internal spinal alignment (ISL) and Cobb angle.

The backshape-to-spine approach, developed by Drerup and Hierholzer [6] and commercially implemented in the DIERS Formetric system, reconstructs the internal spinal alignment (ISL) directly from surface topography. The process begins with establishing a body-fixed coordinate system (Fig 1(1)) based on the automatically localized Posterior Superior Iliac Spines (PSIS) and the Vertebra Prominens [47]. Within this frame, the method automatically identifies the line of symmetry along the back surface [47], which serves as an approximation of the Spinous Process Line (SPL) (Fig 1(2)). The coordinates of the vertebral body centroids, which define the ISL, are subsequently derived using a trigonometric relationship that relates surface trunk rotation (STR) to ISL (Fig 1(3 and 4)):


$$\begin{array}{cc} x_{\text{c},z} & =x_{\text{SP},z}+L(z)\cdot \sin(K\cdot \Theta_{z})\\ y_{\text{c},z} & =y_{\text{SP},z}+L(z)\cdot \cos(K\cdot \Theta_{z})\\ z_{\text{c},z} & =z \end{array}$$ (1)

Here, $\Theta_{z}$ represents the STR angle along the SPL, and *K* serves as a correction factor that accounts for the difference between surface rotation measured on the skin and the actual vertebral rotation. *L*(*z*) denotes the anatomical distance between the spinous process and the vertebral body centroid and can be calculated as follows:


$$\begin{array}{c} L(z)=0.097\cdot (S-0.359(z-z_{C_{7}})) \end{array}$$ (2)

where *S* represents the total spine length ($z_{C_{7}}-z_{S_{1}}$). Finally, the reconstructed ISL is smoothed using analytical functions such as fifth order polynomials and frequency modulated sines (FMS), and the Cobb angle is calculated from the inflection points of the resulting curve in the coronal plane (Fig 1(5)). A detailed description of fundamental principles relating to the implementation of the complete backshape to spine approach by Drerup is provided in Text A2 of S1 Supplementary Information.

### 2.3 From CT image to dataset for backshape-to-spine-approach validation

Generating a validation dataset from CT images involves two distinct steps: extracting 3D surface models of the skin and vertebrae, and measuring relevant anatomical parameters from the extracted surfaces. While the first step utilizes established open-source tools for surface extraction from CT images, the second required the development of a novel algorithm to automatically define vertebral poses in accordance with standard recommendations.

#### 2.3.1 Extraction of skin and vertebrae surfaces from CT data.

The data extraction from CT images focuses on two key anatomical structures: the patient’s skin surface and and the vertebrae from T_1_ to L_5_. Drerup’s method [6] ideally performs spinal analysis between C_7_ and S_1_; however, due to the limited field of view in the CT scans, the present analysis was restricted to the T1 to L5 range.

**Fig 2:**
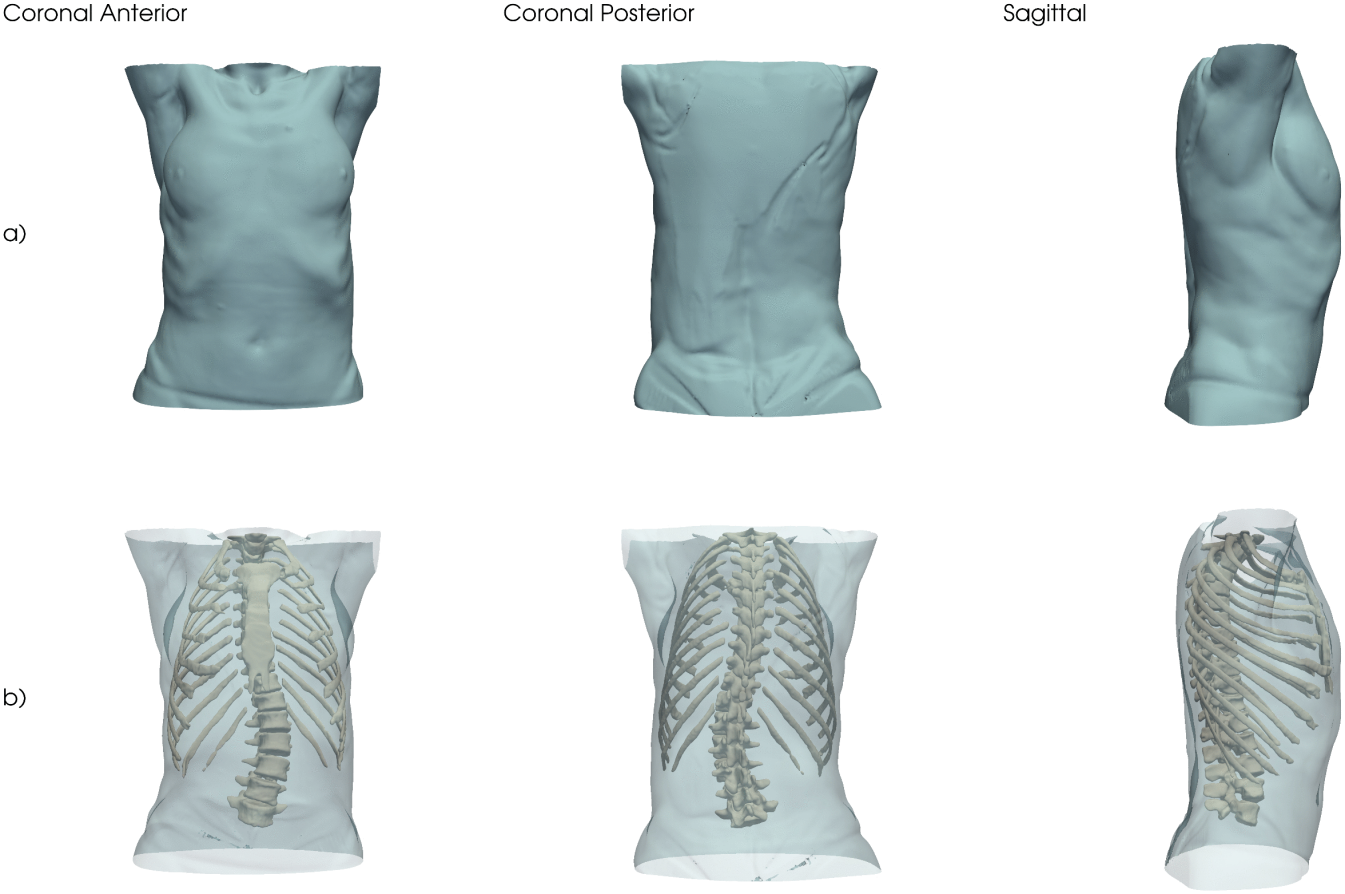
Three-dimensional surface meshes extracted from computed tomography data. (a) Skin surface extraction. (b) Vertebrae extraction. Both structures are depicted in coronal anterior, coronal posterior, and sagittal right views.

For skin contour extraction, we generate a surface mesh based on the CT data that serves as an equivalent to the rasterstereography-derived skin surface used in Drerup’s method [6]. This is accomplished by applying a marching cubes algorithm [48] to the CT scan’s scalar field, using an isosurface threshold that corresponds to the grayscale value of skin (Fig 2 (a)).

Vertebrae extraction requires an additional preprocessing step of individual vertebrae labeling before vertebral surface generation. This was accomplished using deep learning-powered segmentation [49]. The labeled vertebrae were then extracted as surface meshes using the same marching cubes approach (Fig 2 (b)).

#### 2.3.2 Defining vertebra poses from extracted 3D vertebra surfaces.

**Fig 3:**
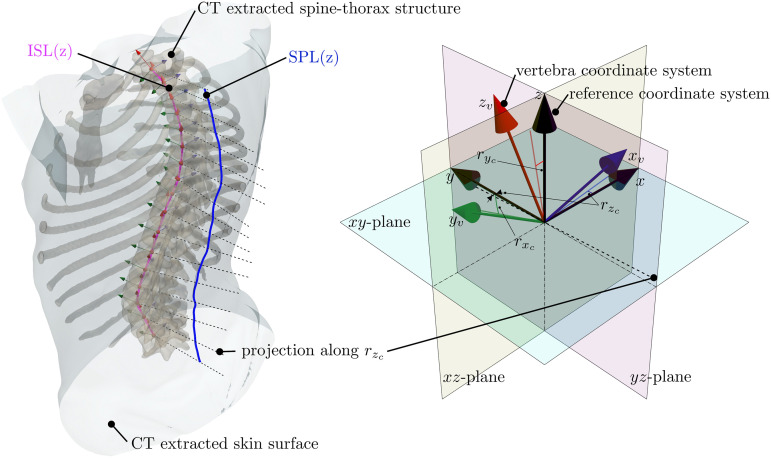
Extracted skin surface, vertebrae, and thorax from CT data, with vertebrae shown alongside their local coordinate systems defining vertebral pose. The intersegmental spinal line (ISL, magenta) connects vertebral centroids, while the spinal process line (SPL, blue) connects the projections of vertebral centroids along the axial rotation in the transverse plane. Right panel illustrates an example vertebra coordinate system $\left(x_{v},y_{v},z_{v}\right)$, the reference CT coordinate system (*x*,*y*,*z*), and the corresponding rotation angles $\left(r_{x_{c}},r_{y_{c}},r_{z_{c}}\right)$.

Vertebral pose is defined by the centroid of the vertebra body, which specifies the vertebra’s spatial location, and by the orientation, which describes its rotations $\left(r_{x_{c}},\;r_{y_{c}},\;r_{z_{c}}\right)$, as illustrated in Fig 3. These rotations indicate how the three axes of the vertebral coordinate system ($x_{c}$, $y_{c}$, $z_{c}$) are oriented relative to a reference coordinate system.

Accurately determining vertebral pose presents significant challenges due to the complex, non-uniform geometry of vertebrae. Conventional approaches typically rely on manually marking landmarks from CT data using 2D cross-sectional analysis [50–52]. These methods are prone to errors, particularly in axial rotation measurements when significant sagittal and/or coronal rotations are present, which is often the case [53], and they suffer from human judgment variability. More sophisticated three-dimensional numerical methods have been proposed [54] which overcome these limitations; however, none of them provide publicly available implementations ready for direct application.

To address these limitations, we present a novel, fully automatic algorithm for vertebral pose extraction based on robust symmetry detection principles. This algorithm is specifically designed to account for the imperfect symmetry of biological structures and remains insensitive to noise and tessellation artifacts common in 3D surface data.

The extraction of vertebral body centroids and vertebral coordinate systems from the vertebrae surface meshes is performed using a fully automatic algorithm, which operates on 3D data in two main steps: symmetry plane estimation and definition of vertebral coordinate systems following the recommendations of the International Society of Biomechanics (ISB) [55,56].

##### Symmetry Plane Estimation.

**Fig 4:**
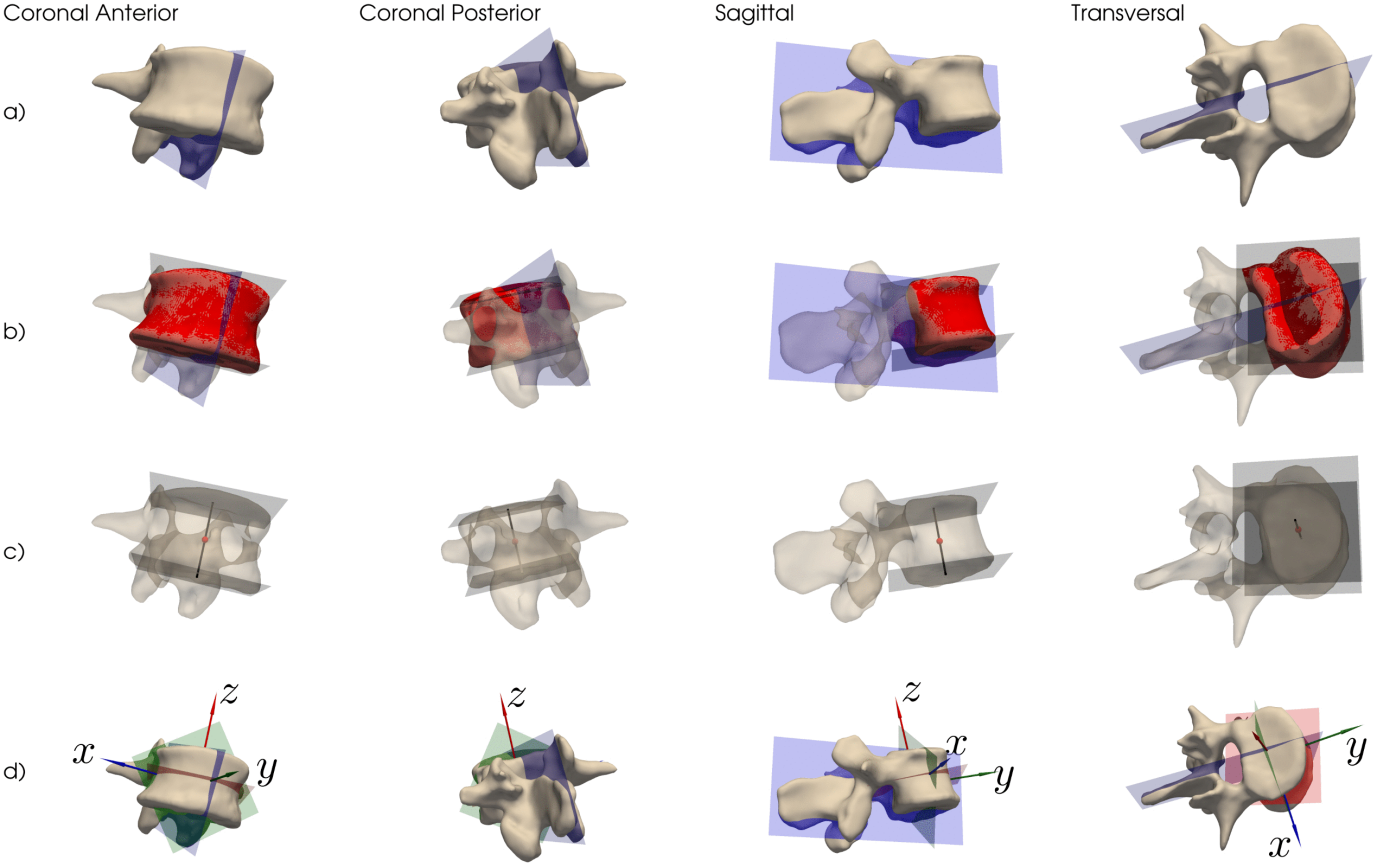
Vertebral pose extraction algorithm: (a) estimation of the symmetry plane (violet plane), (b) segmentation of the vertebral body (red body) and identification of the top and bottom plates (grey planes), (c) definition of the vertebral centroid (red point) as the midpoint of the line connecting the midpoints of the top and bottom plates, and (d) establishment of the local coordinate system (xy-plane: red, yz-plane: violet, zx-plane: green).

Since no vertebra is perfectly symmetric and surface mesh objects inherently contain noise and tessellation dependencies, the algorithm estimates a symmetry plane (violet plane in Fig 4 (a)) using two complementary methods: a symmetry measure established for CT data [54] and a symmetry-finding approach based on generalized moments [57]. The symmetry measure evaluates the difference between the original object and its reflection by calculating the average distance between corresponding points on both surfaces. Ideally, the plane with the lowest average distance indicates the true symmetry plane. However, this approach can yield false positives—where an incorrect plane produces a low average distance—due to natural biological asymmetries (e.g., unequal transverse processes and deformed spinous processes), data noise, or uneven mesh tessellation. The moment-based approach, specifically designed for real objects without perfect symmetry, is robust against noise artifacts and mesh tessellation variations commonly found in scanned or CT-extracted geometries. This approach uses the moment function introduced by [57], which quantifies the spatial asymmetry of a 3D body. The function calculates the sum of the perpendicular distances from each surface face center to an arbitrary symmetry plane passing through the center of mass, weighting each distance by its respective face area. The orientation of this plane is defined by two input angles. Potential symmetry plane candidates are identified by finding minima of the moment function—a necessary but not sufficient condition for symmetry. After these potential candidates for symmetry planes are identified, the symmetry measure is then applied only to these preselected symmetry plane candidates, thereby compensating for the measure’s sensitivity to noise and data imperfections.

##### Definition of vertebral local coordinate system.

Using the identified symmetry plane to isolate the vertebral body, a random sample consensus (RANSAC) algorithm [58] locates the vertebra’s top and bottom plates (grey planes in Fig 4 (b)).

Following ISB recommendations [55,56], the vertebral centroid is defined as the midpoint of the line connecting the geometric centers of the upper and lower endplates (red point in Fig 4 (c)).

While the standard ISB definition aligns the z-axis strictly with the endplate centers, we found this to be unstable for deformed vertebrae. To address this, we developed a novel modified orientation that incorporates the vertebral symmetry plane.

The coordinate system is then constructed as follows (Fig 4 (d)):

1. The **y-axis** is defined as the cross product of the symmetry plane normal and the mean of both endplate normals. Geometrically, this represents the intersection line between the symmetry plane and the average endplate planes.
2. The **z-axis** is calculated as the average of the mean of both endplate normals and the longitudinal vector within the symmetry plane (orthogonal to y).
3. The **x-axis** is determined as the cross product of the y and z axes.

This approach yields a more robust z-axis, particularly in complex and deformed vertebrae, and was validated by an orthopedic specialist through visual comparison of both definitions.

This completes the generation of the CT extracted dataset. By defining the skin surface, vertebral poses, centroids and endplate normals for each vertebra, the dataset now contains all necessary geometric information to derive any spinal and skin surface metric. The subsequent sections will focus specifically on calculating the parameters required to validate our CT extracted dataset using the Drerup’s backshape-to-spine approach [6].

### 2.4 Calculation of CT-derived ground truth parameters

To use Drerup’s backshape-to-spine approach to validate the applicability of our CT-derived dataset, we must first calculate the ground truth values for the key parameters identified in Section 2.2: the continuous parameters ISL, SPL, *L*, and Θ as functions of the vertical coordinate *z*.

Although our vertebra pose extraction algorithm (Sec. 2.3.2) produces a set of discrete poses for each vertebra, the biomechanical constraints of the spine result in these poses naturally following a smooth, continuous path along the *z*-axis. This biological continuity allows us to construct accurate continuous representations along the *z* axis of both the vertebrae centroids (as *ISL*_true_(*z*)) and the individual vertebrae rotations $r_{x_{c,true,z}},\;r_{y_{c,true,z}},\;r_{z_{c,true,z}}$, using cubic Hermite spline interpolation between the discrete vertebral measurements. As a result, the continuous *ISL*_true_(*z*) curve passes exactly through the vertebral centroids and inherits the tilts around the *x*- and *y*-axes $\left(r_{x_{c,true,z}},\;r_{y_{c,true,z}}\right)$ from the individual vertebrae, since its derivatives at the centroids match those of the discrete vertebral orientations (Fig 3). Consequently, this approach yields an accurate and biomechanically plausible continuous representation of the internal spinal line obtained from discrete vertebra poses.

In Drerup’s model, vertebral rotation is approximated by the product of the STR angle Θ and the factor *K* (Sec. 2.2). Consequently, the continuous vertebral rotation profile derived from the CT data defines the ground truth for this estimate. To provide a comprehensive validation, we also compute the ground truth STR angle, $\Theta_{SPL_{true}(z)}$, directly along the true SPL derived from the CT surface. The reference parameter $SPL_{true}(z)$ is derived through geometric projection of vertebral centroid coordinates ($ISL_{true}(z)$) onto the skin surface, following the axial rotations $r_{z_{c},true,z}$ measured from 3D vertebral orientations (Fig 3). The true anatomical distance between the spinous process and the vertebral centroid, $L_{true}(z)$, is then computed as the Euclidean distance between the SPL and ISL curves. The reference Cobb angle, $\alpha_{\text{Cobb,true}}$, is computed according to Cobb’s definition [2] as the angle between the top and bottom plates of the two most inclined vertebrae—those exhibiting the greatest relative rotation around the y-axis—positioned above and below the apex, which is defined as the most laterally deviated vertebra.

### 2.5 Applying our Drerup-based implementation to the CT extracted skin surface

Since Drerup’s method solely uses surface topography, our Drerup-based implementation was applied to our CT-extracted skin surfaces to calculate the estimated parameters for each patient, following the procedure outlined in Section 2.2 using a constant factor *K* = 1. This yields a direct correspondence between the surface-derived estimates and the anatomical ground truth values calculated from the CT data, with the product of the STR angle Θ and the factor *K* being the estimate for the vertebral rotation around the z-axis $r_{z_{c,est,z}}$. The specific parameter pairs used for this comparative validation are summarized in Table 1.

**Table 1:** Parameter pairs used for comparative validation.

	Parameter	estimated	true
Input Parameters	SPL	$SPL_{est}(z)$	$SPL_{true}(z)$
	STR angle	$\Theta_{SPL_{est}(z)}$	$\Theta_{SPL_{true}(z)}$
	SPL-to-ISL distance	$L_{est}(z)$	$L_{true}(z)$
Output Parameters	Vertebra rotations	$r_{z_{c,est,z}}$	$r_{z_{c,true,z}}$
	ISL	*ISL*_est_(*z*)	*ISL*_true_(*z*)
	Cobb angle	$\alpha_{\text{Cobb,est}}$	$\alpha_{\text{Cobb,true}}$

Because the original software for Drerup’s method is not publicly available, we developed an independent implementation based on the primary literature [6,41,47]. To successfully apply this framework to our specific patient cohort, we introduced three necessary adaptations to the standard protocol. First, the standard protocol relies on a body-fixed coordinate system defined by the posterior superior iliac spine (PSIS) and the vertebra prominens (C7). However, soft tissue deformation and age-related skin laxity in our older cohort rendered these surface landmarks unreliable in ten patients. Consequently, all parameters were calculated directly within the intrinsic CT coordinate system. To validate this adaptation, we quantified the alignment between the standard body-fixed frame and the CT coordinate system in the seven patients with clearly identifiable landmarks. This analysis demonstrated a strong correspondence, with average angular deviations below five degrees (Fig A3 in S1 Supplementary Information), confirming the validity of the CT coordinate frame for this analysis. Second, because the PSIS was frequently obscured, we bypassed the automated localization of the PSIS and C7. Beyond defining the body-fixed coordinate system, Drerup’s method utilizes these markers solely to calculate the parameter L (Eq. 2) and to guide the internal criterion for automated symmetry line detection. As the most suitable anatomical substitute, we instead utilized L4 and C7 coordinates that were manually identified and marked by an orthopedic specialist. Third, in one patient with severe thoracic scoliosis, the automated symmetry line identification [47] failed because the extreme lateral curve deviation extended too far beyond the standard longitudinal axis connecting the pelvis and C7. To resolve this, we manually traced a preliminary initialization line approximating the expected symmetry line. The algorithm then successfully identified the final symmetry line points based on this manually adapted initialization.

### 2.6 Validation

The method of automatically extracting datasets for verifying the backshape-to-spine approach is validated through two evaluations: (1) comparing automatically extracted vertebral poses using our algorithm with expert-annotated poses, and (2) applying our implementation of a well-established backshape-to-spine approach (Drerup’s method) on the CT-extracted dataset and comparing the results to existing literature values. The first evaluation assesses whether the extracted values fall within the range of expert annotations, despite their limitations as imperfect ground truth, which is discussed in Section 2.6.1. The second evaluation assesses the applicability of the CT extracted dataset for the validation of our Drerup-based implementation. If applying a well-established method to the extracted skin surfaces and validating the output against the CT ground truth yields results consistent with existing literature, it establishes its suitability for verifying backshape-to-spine approaches.

#### 2.6.1 Comparing automatically extracted vertebra poses with expert-annotated poses.

Validation of automatically extracted vertebral poses is not straightforward. First, established biomechanical protocols, such as the 3D terminology by Stokes [56] and the International Society of Biomechanics recommendations by Wu et al. [55], define abstract spatial coordinate systems for the spine. However, no standardized protocol describes the exact manual anatomical landmark annotations required to implement these definitions.

Second, various methods have been developed to measure individual spinal parameters. For example, approaches by Aaro et al. and Vrtovec et al. successfully measure isolated variables like axial rotation, sagittal inclination, and coronal inclination using specific geometric constructions [59–62]. Despite the availability of these individual measurement techniques, no unified protocol exists to combine them to define a complete 3D vertebral pose.

Third, existing manual approaches [42,52,59] typically evaluate rotation by operating on a single 2D axial cross-section. This simplification introduces geometric projection errors because it fundamentally ignores the out-of-plane tilt—specifically the sagittal and coronal inclinations—of the 3D vertebrae, an issue highlighted by previous symmetry-based studies [45,54,59].

Finally, our automatic approach calculates the vertebral symmetry plane by considering the entire vertebral geometry, which is impossible to replicate manually. Because human measured reference methods are restricted to discrete landmarks, observed deviations between the methods do not automatically imply inaccuracy in the automatic method. Rather, they highlight the discrepancy between a sparse landmark-based approximation and a holistic geometric calculation.

To address these issues, we developed a semi-automatic protocol that allows practitioners to mark vertebral CT data in 3D. The four points required to define the full vertebral pose using the ISB protocol are the midpoints of the bottom and top vertebral plates to define the vertebral body centroid and z-axis, and two similar landmarks at the bases of the right and left pedicles to define vertebral axial rotation and thus the y-axis. Since the exact landmarks are not described in [55,56], we used a well-validated method [60] established by Aaro and Dahlborn [59], which identifies the dorsal central aspect of the vertebral foramen that, together with the vertebral body centroid, defines the y-axis direction. Since, as mentioned earlier, no standardized protocol exists for manually defining the vertebral symmetry plane, the landmark-based approach represents the closest feasible method to obtain comparable results between manually annotated and automatically extracted vertebral poses.

To accurately identify the midpoints of the top and bottom vertebral plates, practitioners mark the top and bottom vertebral plates in 3D, and the midpoints are then calculated from the segmented surfaces. In the next step, the vertebra is oriented automatically so that the newly defined z-axis points toward the practitioner, effectively eliminating errors in landmark identification of the dorsal central aspect of the vertebral foramen caused by coronal and sagittal vertebral inclination. In this way, the semi-automatic protocol avoids influencing the practitioner while taking over tasks unsuitable for manual estimation. The user simply marks distinct anatomical landmarks, and the computer automatically calculates skeletal coordinates, such as vertebral endplate centers, which would be highly prone to error if estimated manually.

To validate the automatically extracted vertebral poses, we compared them using the mean absolute error (MAE) calculated from manual annotations performed by three experts — a computational engineer (1) experienced in vertebral anatomy, a mathematician (2) with experience in medical image analysis, and a senior orthopedic specialist (3). Vertebrae from 17 patients were presented in consecutive order, and all annotations were completed in a single session. Prior to annotation, all experts conducted a practice session on one patient’s vertebrae (T_1_ to L_5_) to minimize errors from unfamiliarity with the method.

#### 2.6.2 Evaluating CT dataset applicability for the validation of backshape to spine approaches using our Drerup-based implementation.

In this section, we use the extracted parameters summarized in Table 1 to calculate error metrics of the estimated values obtained from applying our Drerup-based implementation to the CT-extracted skin surface and the true values calculated from the CT data. For quantitative assessment, root mean square error (RMSE), mean absolute error (MAE) and standard deviation (STD) are used as metrics to evaluate deviations between estimated and true parameter values.

The error metrics are chosen so that they are comparable with previous validation studies of Drerup’s method, which are summarized and grouped according to types of ground truth parameters in Table 2.

**Table 2:** Summary of validation studies of Drerup’s method. The table presents MAE and STD of Cobb angle, vertebral rotation and SPL(coronal) as well as RMSE for axial vertebra rotation and ISL(coronal). “Th” and “Lu” indicate that the results belong to the thoracic and lumbar curve regions, respectively. The categories “All,” “Mild,” and “Med./Sev.” refer to all types of spine curves, mild curves, and moderate to severe curves, respectively.

Ground truth	Work	Cobb angle (MAE, STD)	Vertebra rotation RMSE	ISL RMSE	SPL (MAE,STD)
2D-Xray	[6]	(—, 7.9)°	3.8°	4.6 mm	—
	[41]	—	2.7°	3.9 mm	—
	[37]	Th:(5.8, 2.5)° Lu:(8.8, 2.6)°	— —	— —	— —
	[38]	(19, 11)°	—	—	—
	[39]	All:(6.4, 4.7)° Mild:(4.3, 3.5)° Med. /Sev.:(9.7, 4.7)°	— — —	— — —	— — —
	[63]	—	7.9°	—	—
	[23]	—	6.7°	3.2mm	—
	[64]	—	5.3°	6.4mm	—
3D-Xray	[39]	All:(7.5, 6.4)° Mild:(5.2, 3.9)° Med. /Sev.:(11.2, 7.9)°	— — —	— — —	— — —
Manual	[65]	—	—	—	(5.8, 2.28)mm

To ensure a robust evaluation, we define the estimated Cobb angles as non-interpretable if the identified apex deviates from the true apex by more than one vertebral level, thus excluding anatomically implausible results.

At first, the deviation between estimated and true values of input and output parameters are analysed for each individual patient. Then, RMSE, MAE and STD are calculated for the continuous parameters ISL, SPL, *L*, and Θ, by evaluating the functions along the spine at each true vertebral position from T1 to L5. For the Cobb angle, the absolute deviation between estimated and true values is reported for individual patients, and the MAE and STD of these deviations are calculated across all patients.

To enable comparison with prior work, RMSE is reported for ISL and axial rotation, while MAE and STD are presented for the remaining parameters.

To demonstrate the full utility of the CT-extracted dataset, a comprehensive analysis of parameter dependencies and error propagation in Drerup’s approach was conducted. As this detailed analysis falls outside the primary scope of this paper, the complete methodology and results are provided in the Text A4 in S1 Supplementary Information.

## 3 Results

This chapter, first presents the deviations between expert-annotated and automatically extracted vertebral poses. Next, we evaluate the deviations between the parameters estimated using our Drerup-based implementation (ISL, SPL, axial vertebral rotation $r_{z_{c}}$, and Cobb angle) and the CT-derived ground truth parameters. For the ISL, SPL, and vertebral rotation, deviations are first averaged per patient to compute the overall cohort mean and standard deviation. For the Cobb angle, the mean absolute error (MAE) and standard deviation are reported for the entire dataset. The SPL-to-ISL distance and STR angle are excluded from this comparison due to a lack of reference values in the existing validation literature.

### 3.1 Comparison between expert annotated and automatically extracted vertebra poses

Table 3 presents the deviations between expert-annotated and automatically extracted vertebral poses. The similarity in MAE and STD values indicates consistent performance among annotators. Good agreement is observed between manual and automatic poses for the ISL parameter, while larger deviations are noted in vertebral rotations. Specifically, sagittal inclination ($r_{x_{c}}$) exhibits greater deviation compared to axial rotation and coronal tilt ($r_{y_{c}}$ and $r_{z_{c}}$), which show comparable deviations.

**Table 3:** Mean absolute error (MAE) and standard deviation (STD) of vertebra body centroids and vertebral rotations around the *z*, *y*, and *x* axes ($r_{z_{c}}$, $r_{y_{c}}$, $r_{x_{c}}$) across all patients. Annotators 1, 2, and 3 are the computational engineer, the mathematician, and the orthopedic specialist, respectively.

Annotator	vertebra body centroid (MAE, STD)	$r_{z_{c}}$ (MAE, STD)	$r_{y_{c}}$ (MAE, STD)	$r_{x_{c}}$ (MAE, STD)
1	(0.17, 0.18) mm	(1.82, 2.20) °	(1.82, 2.03) °	(2.61, 3.05) °
2	(0.20, 0.23) mm	(1.77, 2.07) °	(1.80, 2.02) °	(2.45, 2.89) °
3	(0.25, 0.26) mm	(1.99, 2.21) °	(1.93, 2.25) °	(2.44, 2.81) °

### 3.2 Deviations of our drerup-based implementation on CT-extracted skin surfaces

**Fig 5:**
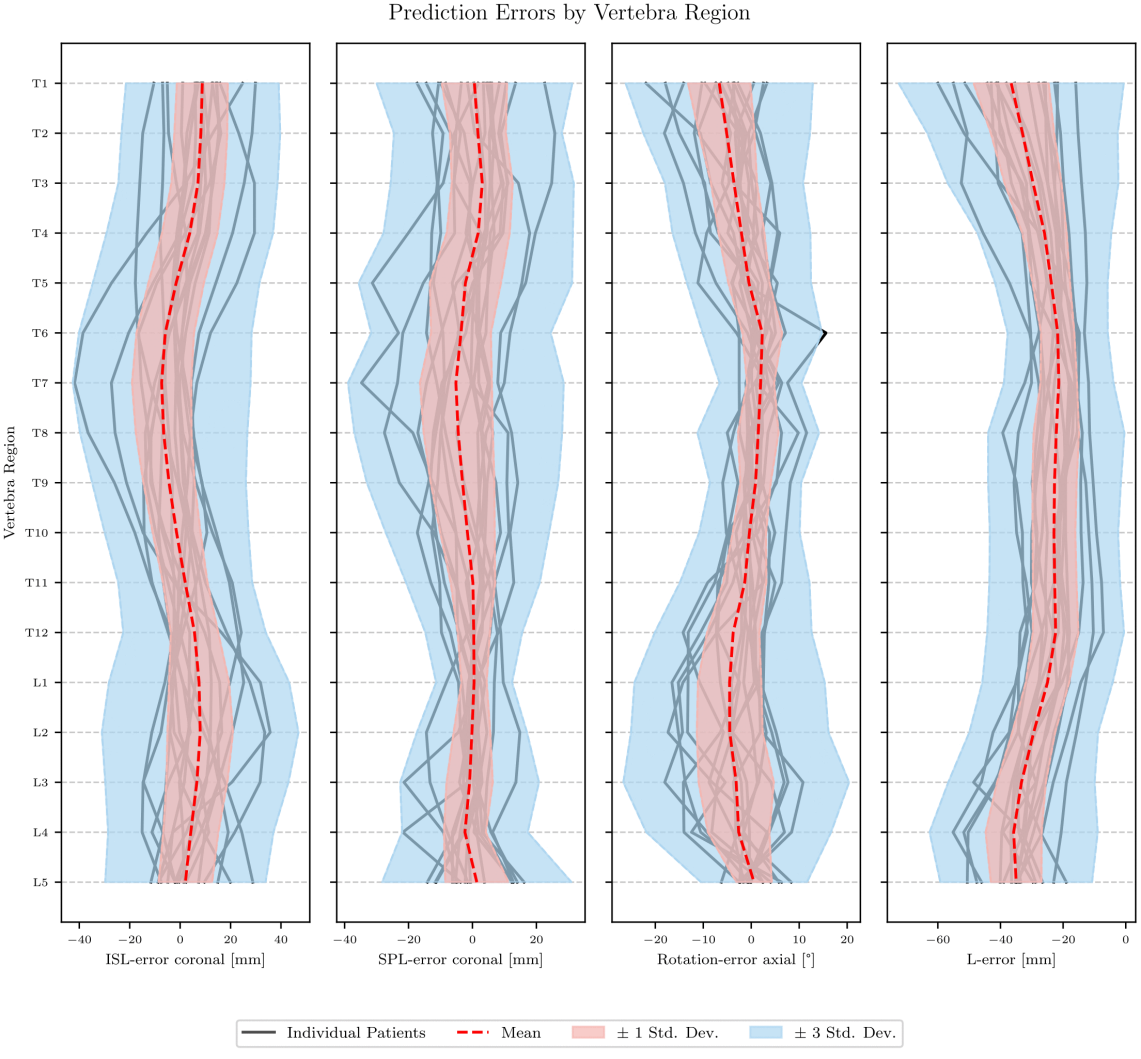
Deviations of SPL, ISL axial vertebral rotation and L between our Drerup-based implementation applied to CT-extracted skin surfaces and CT ground truth across vertebral levels. The plots display individual patient data alongside the overall mean, ±1 and ±3 standard deviation.

Fig 5 details the deviations between the estimates (SPL, ISL, axial vertebral rotation and L) of our Drerup-based implementation applied to the CT-extracted skin surfaces and the CT ground truth parameters across all vertebral levels. The mean deviations for parameters (SPL, ISL and axial vertebral rotation) range around near zero, indicating an absence of systematic bias, while the L parameter shows a large negative bias. The results show high consistency across the cohort, with all individual trajectories falling within ±3 standard deviations of the mean.

**Fig 6:**
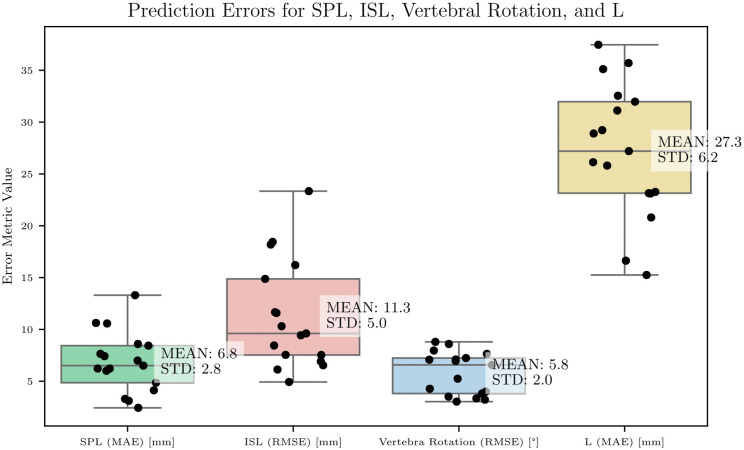
MAE of SPL and L, and RMSE of ISL and axial vertebra rotation evaluated from vertebral levels T_1_ to L_5_ for each patient.

The MAE of SPL and L, as well as the RMSE of ISL and axial vertebra rotation calculated for each patient across vertebral levels T_1_ to L_5_ are shown in Fig 6. The mean MAE of SPL is 6.8 mm with a standard deviation of 2.8 mm, and the mean RMSE of axial vertebra rotation is 5.8° with a standard deviation of 2.0°. Both metrics show a relatively compact error distribution. In contrast, the mean RMSE of ISL is notably higher at 11.3 mm with a standard deviation of 5.0 mm. The ISL distribution shows significantly larger variance, including a prominent extreme value near 24 mm. The parameter L exhibits the largest errors, with a mean MAE of 27.3 mm and a standard deviation of 6.5 mm.

**Fig 7:**
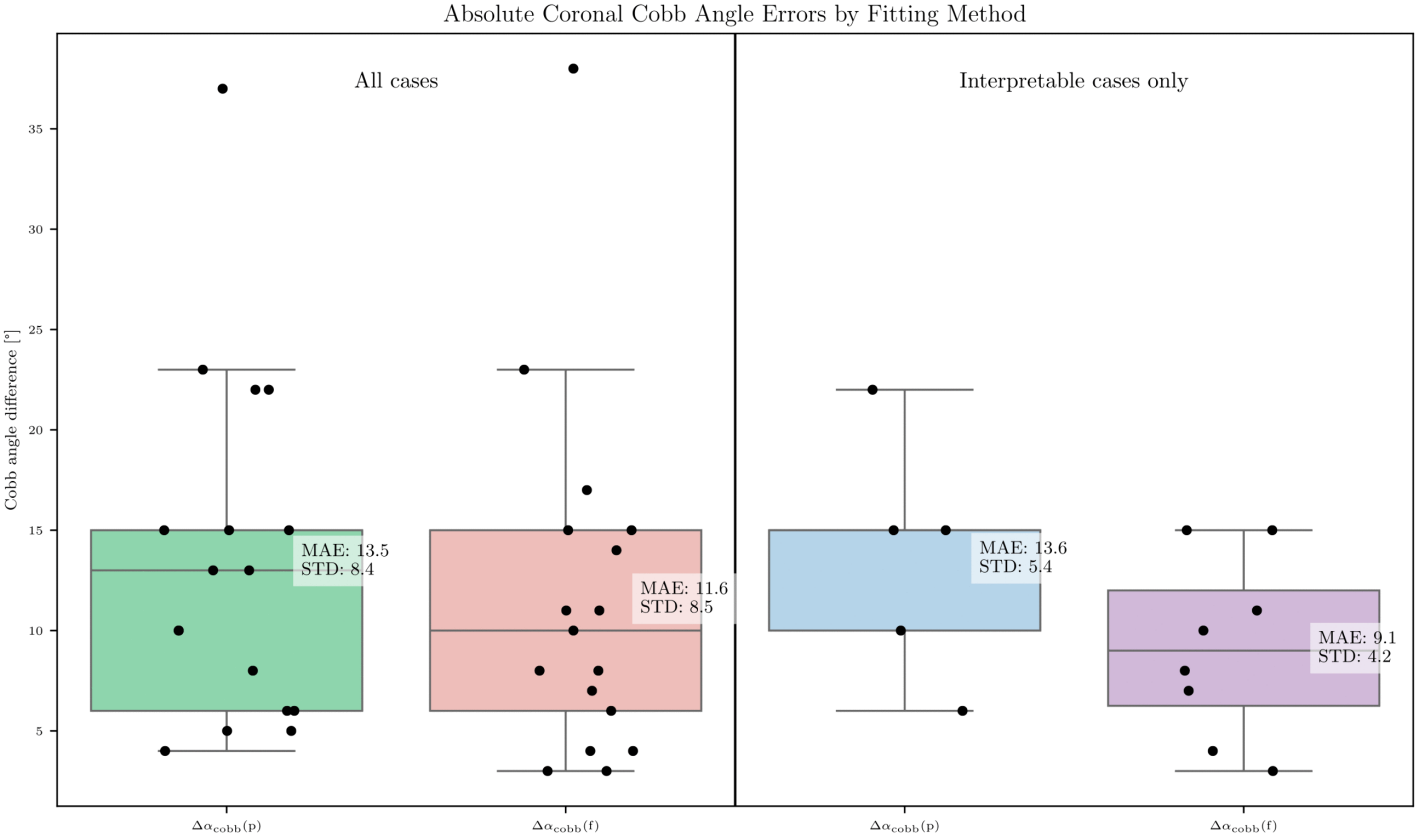
Absolute deviations of coronal Cobb angle across the patient cohort for polynomial $\Delta \alpha_{cobb}(p)$ and FMS $\Delta \alpha_{cobb}(f)$ fitting methods. The left panel: unrestricted cohort. The right panel: restricted cohort, obtained by excluding non-interpretable estimates of Cobb angle, defined as apex deviations exceeding one vertebral level from the anatomical reference established via the standard radiographic Cobb method.

The absolute errors of the coronal Cobb angle estimated using two fitting methods (described in detail in Text A2 in S1 Supplementary information), separated by the interpretability criterion defined in Sec 2.6.2 are presented in Fig 7. When considering all cases, the FMS fitting method ($\Delta \alpha_{cobb}(f)$) results in a lower MAE of 11.6° with a standard deviation of 8.5°, compared to the polynomial fit ($\Delta \alpha_{cobb}(p)$) which has an MAE of 13.5° and a standard deviation of 8.4°. Both error distributions in the unrestricted cohort show large variance and contain extreme outliers approaching 38°.

Excluding non-interpretable cases (apex deviations exceeding one vertebral level) significantly reduces the variance and eliminates extreme outliers across both fitting methods. In this restricted subset, the polynomial fit shows a similar MAE of 13.6° as when considering all cases but with a narrower standard deviation of 5.4°. The FMS fitting method demonstrates the highest overall accuracy on interpretable cases, reducing the MAE to 9.1° with a standard deviation of 4.2° and keeping all absolute errors at or below 15°.

## 4 Discussion

In this study, we presented a novel method for generating a dataset that synchronizes skin surface and vertebral pose information from CT imaging of 17 patients with various spinal deformities. Validation of the CT-extracted dataset was performed by comparison with expert-annotated vertebral poses, as well as by applying our Drerup-based implementation as reference of a well established backshape-to-spine approach [6]. This discussion first contextualizes the deviations between expert-annotated and automatically extracted vertebral poses. Next, we compare the performance of our Drerup-based implementation on CT-extracted skin surfaces against existing literature. Finally, we evaluate the clinical implications of utilizing CT-extracted datasets and outline directions for future research.

### 4.1 Comparison with expert annotations

Deviations between expert-annotated and automatically extracted vertebral poses can be directly attributed to several specific factors. The deviation in the vertebral body centroid appears solely due to differences in endplate segmentation between expert and automatic methods, as the underlying approach for centroid calculation is identical in both methods. These differences are minimal, as reflected by small MAE values of 0.17 mm, 0.20 mm, and 0.25 mm for annotators (1), (2), and (3), respectively (see Table 3).

No significant differences were observed between annotators, as their MAE values fall within each other’s standard deviation ranges, as summarized in Table 3. This consistency suggests that the manual pose extraction protocol is robust and reliable.

Two primary factors that contributed to deviations in vertebral rotations are differing definitions of the z-axis and y-axis. For the z-axis, expert annotations strictly followed the ISB recommendations [55,56], which, as confirmed by an orthopedic specialist reviewing visual comparisons, sometimes resulted in a z-axis orientation that does not visually align with the vertebral orientation. In contrast, our automatic method defines the z-axis more robustly by incorporating the full information of both vertebra endplates and the symmetry plane.

Regarding the y-axis, expert annotations rely on a single landmark at the dorsal central aspect of the vertebral foramen, whereas the automatic method defines the y-axis based on the overall symmetry of the vertebral body. This difference in definition resulted in angular deviations with MAE values ranging from 1.77° to 2.61° across all vertebra rotations and annotators.

### 4.2 Comparison with literature

For the SPL in the coronal plane, averaged across all patients the MAE of 6.8 mm and STD of 2.8 mm (Fig 6) aligned with literature values MAE of 5.8 mm and STD of 2.28 mm (Table 2). Similarly, axial vertebra rotation was estimated with the average RMSE of 5.8° and STD of 2.0° over all patients (Fig 6), which is consistent with the range reported in prior studies (RMSE: 2.7°–7.9°, Table 2). These results suggest, that our Drerup-based implementation can be successfully applied to CT extracted skin surface, because the SPL and STR error margins align with those reported in previous studies that used conventional 3D surface scans and reference radiographs.

The parameter *L* exhibited deviations with an overall MAE across all patients of 27.3 mm and STD of 6.2 mm (Fig 6) that unfortunately cannot be directly compared to literature due to the absence of prior validation studies. These errors are predominantly systematic, as evidenced by the notable bias in the deviations across all patients in Fig 5, likely stemming from demographic discrepancies and our necessary methodological adaptation of using the L4 marker instead of S1. The linear relationship used for the estimation of *L* in Eq. 2 was originally derived for AIS patients [6] –a population typically comprising children and adolescents [1]– while our dataset included adult spinal deformities. Additionally, the anatomical distance from L4 to C7 is inherently shorter than the standard distance from S1 to C7, meaning this substitution naturally results in the observed systematic underestimation of *L*.

The average RMSE of 11.3 mm and STD of 5.0 mm of the estimated ISL in the coronal plane across all patients (Fig 6) was higher than the largest deviation with an RMSE of 6.4 mm reported by [64] (Table 2). Unfortunately direct statistical comparison is impossible due to absent patient-to-patient standard deviations in prior studies. The higher average RMSE observed here can be explained by two main factors: first, the large errors in *L* with an MAE of 27.3 mm (Fig 6); and second, the use of a synchronized dataset, which for the first time directly links the reference ISL and back surface. This increased methodological precision likely reveals error components that previous studies, which did not use synchronized data, could not capture, explaining the remaining difference above literature values.

The average MAE across all patients of the estimated coronal Cobb angle (Fig 7) of 13.5° with an STD of 8.4° using polynomial fitting and 11.6° with an STD of 8.5° using FMS fitting, were consistent with literature values (MAE of 11.2° and STD of 7.9° for 3D-Xray, Table 2).

The consistent alignment with literature values of the deviations for estimated SPL, axial vertebral rotation, and Cobb angle derived from our CT-based dataset using Drerup’s established backshape-to-spine approach [6], demonstrates the validity of using CT images to extract perfectly synchronized skin and vertebral data. This provides a reliable alternative for the verification of backshape-to-spine approaches, unlike the traditional combination of surface scans and 2D X-rays, which suffers from posture-induced alignment errors.

Beyond the primary validation, the extended analysis of methodological discrepancies and parameter dependencies in Text A4 in S1 Supplementary Information showcases our dataset’s capacity for detailed error tracking in future algorithmic development.

### 4.3 Limitations of the study and future work

Taken together, the presented results demonstrate that our method enables the derivation of a synchronized back surface and spine dataset from CT images, opening up the way to a reliable foundation for the verification of back-shape to spine approaches. However, several limitations of the present study must be mentioned.

First, a direct, isolated validation of our Drerup-based implementation—which was used to verify the applicability of our CT-based dataset—was not feasible. Such an isolated validation would necessitate reference data, such as the original datasets (or equivalent back-surface and X-ray cohorts) used in other validation studies, or pairs of commercial surface scan and CT scan from the same patients. This creates a coupled scenario where errors cannot be strictly isolated between our Drerup-based implementation and the variance introduced by our novel CT extracted dataset. However, we validated the system holistically: applying our implementation to our CT extracted data yielded error margins within reported ranges from literature. This consistency strongly supports both the accuracy of our implementation and the study’s main scope, the applicability of the CT data, despite the inability to perfectly decouple their individual error contributions. Second, all patients were scanned in the supine position, which leads to a different spinal alignment compared to the standing posture. This discrepancy may result in significant differences in Cobb angle measurements between supine and standing positions [66]. However, previous studies have shown that axial vertebral rotation remains largely unaffected by positional changes [59]. This suggests that, because the back surface and spine are captured simultaneously in a CT scan, any posture-induced spinal deformation is accompanied by corresponding changes in skin surface geometry. This synchronized adaptation preserves the fundamental relationship between the two structures. Consequently, analytical backshape-to-spine approaches like the one applied in this study, which rely on these geometric relationships, maintain their methodological validity despite the postural difference. However, the postural difference carries distinct clinical implications, as treatment decisions are strictly based on standing curve magnitudes. Statistical models trained exclusively on supine data would systematically underestimate curve severity if applied directly to standing patients. Therefore, any future clinical application of statistical algorithms developed using this dataset will require subsequent calibration or domain adaptation steps to translate supine-validated predictions into actionable, standing-equivalent metrics. Such adaptations are highly feasible given the established linear relationships between supine and standing measurements reported in the literature [67,68]. Third, direct contact between the back surface and the CT table can cause localized flattening of the skin, particularly in areas such as the hips and shoulder blades. While this effect was minimal in the lean patients of this cohort and did not impact SPL or rotation estimates, the obscuration of PSIS landmarks in individuals with higher body fat or age-related skin laxity represents a notable limitation.

Fourth, the limited sample size reduces the statistical power of the analysis, preventing a separate analysis of different scoliosis types and severity levels. Finally, the patients’ age range (54–88 years) does not correspond to the typical age range for adolescent idiopathic scoliosis (AIS) patients (10–18 years) [1]. Beyond limiting the direct generalizability of our findings to the AIS population, age-related skin laxity within this older cohort rendered standard anatomical landmarks unreliable in 10 patients. This forced a deviation from standard ISB recommendations, necessitating the use of the intrinsic CT coordinate system rather than a standard body-fixed coordinate system. However, within the context of a proof-of-concept validation, this skin laxity effectively served as a worst-case scenario. The observed error margins were comparable to the corresponding values from existing literature despite this tissue-level confounder, which demonstrates the robustness of our dataset generation method.

Based on the results of the present study, several directions for future work are planned to strengthen the validity and application of our approach. First, we will expand the patient cohort and CT extracted dataset to improve the statistical power and generalizability when used for STAM verification. Alongside this, we will also utilize Magnetic Resonance Tomography (MRT) scans, as outlined in the methods section 2, since the method requires only surface meshes of the vertebrae and skin, which can also be obtained from MRT imaging. Our initial reliance on a CT-based dataset rather than MRT was strictly driven by the lack of robust automated MRI segmentation tools during our study design in early 2024. Comprehensive whole-body MRI extraction tools, such as TotalSegmentator MRI, were not officially published until February 2025 [69], making their integration chronologically unfeasible for the current study. However, applying our approach to MRT data in the future not only increases the patient cohort size but, more importantly, allows inclusion of younger patients, as MRT scans are available for this group. Thus, we can extend the diversity of the patient cohort to better represent the targeted AIS population.

Finally, we believe that this work establishes a robust foundation for future research by providing a synchronized, CT-derived dataset that overcomes the limitations of unsynchronized back surface and spine alignment data as it was previously used [6,23,37–39,41,63–65]. Furthermore it enables reference measurements for all relevant parameters due to the comprehensive nature of CT imaging. The integration of validated biomechanical modeling with synchronized data will enable more precise error attribution to the analytical method itself, rather than to uncertainties in the underlying dataset, and will facilitate the development of next-generation, patient-specific analytical tools for spinal deformity assessment.

## 5 Conclusion

This study demonstrates a novel methodology for automatically generating synchronized datasets of skin surface and vertebral pose information from computed tomography images, employing a newly introduced automated symmetry-based vertebra pose detection algorithm that adheres to current clinical recommendations. The automatically extracted vertebra poses were validated against expert annotations and showed high agreement. Moreover, comprehensive comparison between parameters estimated using our Drerup-based implementation applied to CT-extracted back surfaces and CT-derived ground truth values was performed. The observed STD ranges of the deviations between estimated and true key parameters, such as SPL, axial vertebral rotation, and Cobb angles, did not exceed critical values from the literature. This observed agreement confirms our primary hypothesis, that CT-extracted skin surfaces combined with automated vertebral poses extraction, may serve as a valid alternative to conventional surface topography and radiographic data for validating backshape-to-spine approaches.

Key contributions of this work include the use of computed tomography imaging for highly accurate three-dimensional information, automated extraction of synchronized skin surface and vertebral pose data that eliminates human errors. This synchronization overcomes a major limitation of previous datasets, where differences in acquisition timing and patient posture hindered reliable method validation, misattributing any observed deviations during verification to the analytical method itself, rather than to inconsistencies in the dataset.

However, the limited sample size restricts statistical power and direct contact with the computed tomography table may locally distort skin surface geometry, particularly in non-lean patients, which could affect other surface measurements beyond those examined in this study.

Future research should focus on expanding the patient cohort using additional CT as well as MRT resources, since, in principle, the presented algorithm relies solely on extracted surface meshes of the vertebrae and skin, which can be obtained from both imaging modalities.

In conclusion, despite the mentioned limitations, this study provides a robust foundation for future analytical method development and verification, offering a pathway toward more accurate non-radiographic evaluation of spinal deformities.

## Supporting information

S1 File Supplementary Information. (PDF)
