# Supplementary material for: Automated CT dataset generation as a novel concept for verification of backshape-to-spine approach and Cobb Angle Estimation
Source: PLoS One. 2026 Jul 17;21(7):e0353213. doi: 10.1371/journal.pone.0353213 (PMC13379039; doi:10.1371/journal.pone.0353213)
Supplement: S1 File — (PDF) [file pone.0353213.s001.pdf]

S1: Supplementary Information

David Fräulin[1], Irina Sidorenko[2], Renée Lampe[3,*]

1 Department of Clinical Medicine, Center for Digital Health and Technology, Orthopedic Department, Research Unit for Paediatric Neuroorthopedics and Cerebral Palsy of the Buhl-Strohmaier Foundation, Klinikum rechts der Isar, School of Medicine and Health, Technical University of Munich, Ismaninger Str. 22, 81675 Munich, Germany, david.fraeulin@tum.de
2 Department of Clinical Medicine, Center for Digital Health and Technology, Orthopedic Department, Research Unit for Paediatric Neuroorthopedics and Cerebral Palsy of the Buhl-Strohmaier Foundation, Klinikum rechts der Isar, School of Medicine and Health, Technical University of Munich, Ismaninger Str. 22, 81675 Munich, Germany, sidorenk@ma.tum.de
3 Department of Clinical Medicine, Center for Digital Health and Technology, Orthopedic Department, Research Unit for Paediatric Neuroorthopedics and Cerebral Palsy of the Buhl-Strohmaier Foundation, Markus Würth Professorship, Klinikum rechts der Isar, School of Medicine and Health, Technical University of Munich, Ismaninger Str. 22, 81675 Munich, Germany, renee.lampe@tum.de

* renee.lampe@tum.de

Appendix

A1 Table

highthoracic spinal curve	lumbar mild	lumbar moderate	thoracolumbar moderate	thoracic moderate	highthoracic moderate	lumbar severe	thoracic severe
P1	P2, P3, P4, P5	P6, P7, P8, P9	P10	P11, P12, P13, P14	P15	P16	P17

Patient distribution across spinal region and severity cases

A2 Text

1 Fundamentals

This section describes the underlying principles, mathematical formulations, inherent assumptions, and component algorithms of the widely cited backshape-to-spine approach developed by Drerup and Hierholzer, which has been commercially implemented in the DIERS Formetric 4D system. Although this methodology has been described in several publications [1–3], we believe that presenting it in the style of an implementation guide will help simplify the concepts for the reader.

1.1 Reference coordinate system

The use of a body-fixed reference coordinate system is of crucial importance for many biomechanical measurements, as meaningful interpretation of the final results depends on the choice of an appropriate reference system into which the data must be transformed [4]. The standard recommendation by the International Society on Scoliosis

Orthopaedic and Rehabilitation Treatment (SOSORT) [5] proposes defining landmarks that are easily accessible in humans through external palpation or estimation methods, using the skin overlying the posterior superior iliac spine dimples (PSIS) as reference landmarks. While several interpretations of this recommendation exist, the most common, referring to [1, 6], is as follows. As illustrated in main text Fig 1(1), the reference coordinate system is defined with its origin located at the midpoint between the PSIS dimples. The z axis is directed upwards, passing through the midpoint between the dimples and through the vertebra prominens. The x axis is directed to the right side and perpendicularly to the z axis, lying in the plane through the vertebra prominens and both dimples. The y axis is directed forwards and perpendicularly to the x and z axes.

1.2 Estimation of internal spinal alignment (ISL)

Based on the assumption that the surface topography of the skin accurately represents the underlying spinal deformity, the methods of [1–3], as illustrated in main text Fig 1, predict the three-dimensional ISL. The ISL is defined as the continuous trajectory of x- and y-coordinates ($x_{c,z}$, $y_{c,z}$) for z-values along the z-axis passing through vertebral body centroids between the z coordinates z_{C_7} and z_{S_1} of the vertebrae C_7 and S_1: $\mathrm{ISL}(z) = [x_{c,z}, y_{c,z}, z]^T, \quad \forall\, z \in [z_{C_7}, z_{S_1}]$. The x- and y-coordinates are calculated using the following steps:

1. **Define the body-fixed coordinate system:** As described in Section 1.1, the first step is to define a body-fixed coordinate system that provides the essential reference frame for all subsequent calculations. Two primary approaches exist for defining this system: Turner-Smith et al. manually mark the PSIS dimples and vertebra prominens on the patient's skin [2], while Drerup developed an automatic algorithm to locate these anatomical landmarks [4].

2. **Estimate spinous process (SP) positions:** The next step involves estimating SP positions along the continuous spinous process line (SPL), also referred to as the symmetry line (main text Fig 1(2)). This line is defined as the three-dimensional trajectory of x- and y-coordinates ($x_{SP,z}$, $y_{SP,z}$) for z-values along the posterior median furrow of the back surface between the z coordinates z_{C_7} and z_{S_1} of the vertebrae C_7 and S_1:
$\mathrm{SPL(z)} = [x_{SP,z}, y_{SP,z}, z]^T, \quad \forall\, z \in [z_{C_7}, z_{S_1}]$. The SPL can be determined automatically using curvature analysis of 3D back surface scans [4] or manually by marking SP landmarks and fitting an analytical function to these points [2].

3. **Calculate surface trunk rotation (STR) angle:** Next, the continuous STR angle, $\Theta(z), \quad \forall\, z \in [z_{C_7}, z_{S_1}]$, is calculated (main text Fig 1(3)). This angle is defined as the angle between the x-axis of the reference coordinate system and a line connecting two points, A and B, located on either side of the SP point at a distance P (defined as $\frac{1}{10}$th of the distance from vertebra T_1 to vertebra L_4) apart [2]. Note that while several definitions of STR exist, most differ primarily in how points A and B are defined; however, [1] uniquely defines the STR using surface normal directions at SP points, rather than relying on explicit A and B point locations.

4. **Estimate distance L and factor K:** Further (main text Fig 1(4)), calculating the estimated vertebral centroids requires two statistically-estimated parameters: L – the Euclidean distance in the x, y-plane between the SPL and ISL, and the adjustment factor K. For L, two main approaches exist. The first assumes linear

distance changes along the spine, expressed as:

$$L(z) = \begin{cases} 0.097 \cdot (S - 0.359(z - z_{\mathrm{C}_7})), & [1] \end{cases} \tag{1}$$

where S represents the total spine length $S = z_{\mathrm{C}_7} - z_{\mathrm{S}_1}$. The second approach uses vertebra-specific average values derived from anatomical studies [7–9].

The factor K serves as a correction factor that accounts for the difference between surface rotation measured on the skin and the actual vertebral rotation. This constant incorporates anatomical variations such as muscle thickness, rib cage influence, and vertebral morphology. The value of K varies across studies [1–3], partly due to differences in how the STR angle is defined and measured in each method.

5. **Calculate vertebral centroid coordinates:** Finally, with all necessary parameters estimated, the ISL is calculated using the following formulas:

$$\begin{aligned} x_{\mathrm{c},z} &= x_{\mathrm{SP},z} + L(z) \cdot \sin(K \cdot \Theta_z) \\ y_{\mathrm{c},z} &= y_{\mathrm{SP},z} + L(z) \cdot \cos(K \cdot \Theta_z) \\ z_{\mathrm{c},z} &= z \quad \forall\, z \in [z_{\mathrm{C}_7}, z_{\mathrm{S}_1}] \end{aligned} \tag{2}$$

1.3 Estimation of the Cobb angle based on ISL

The ISL provides a radiation-free estimate of spinal alignment comparable to conventional radiographic assessment. However, the Cobb angle, as measured from a radiograph, is not directly available from the ISL. This is because radiographs provide the additional information of vertebral endplates, which radiographic methods use to identify the most tilted vertebrae and measure the angle between them to determine the Cobb angle. To overcome this limitation, Drerup's method uses analytical functions $f(z)$, such as polynomials [2] and frequency modulated sines (FMS) [10], which smooth the estimated ISL via curve fitting, and provide an analytical representation of the ISL in the coronal plane. The method then relies on the assumption that the function's inclination points represent the positions of the most tilted vertebrae and that the slopes of the function at these points represent their inclination. As shown in main text Fig 1(5), functional analysis is then used to obtain the inflection points (ip) (where $f''(z_{\mathrm{ip}}) = 0$, located on either side of the apex, the point of maximum amplitude where $f'(z_{\mathrm{ap}}) = 0$). The Cobb angle is then calculated as the angle between the two normals (Eq. 3), defined by the slopes of the fitted function at the two inflection points, $m_i = f'\big(z_{\mathrm{ip}}\big) \quad \forall\, i \in \{1, 2\}$.

$$\alpha_{\mathrm{Cobb}} = |\arctan(m_1) - \arctan(m_2)| \tag{3}$$

In conclusion, Drerup's method operates by first automatically estimating the SPL from back surface topography. It then calculates the angle Θ along the SPL, derived from the back shape; combined with the factor K, this provides an approximation of the vertebral axial rotation ($r_{z_c,\mathrm{est}}$). Using the estimated distance L_{est} between the SPL and the ISL, Drerup's method reconstructs the ISL, which is subsequently smoothed using analytical functions to determine vertebral inclination in the coronal plane ($r_{y_c,\mathrm{est}}$). This smoothed ISL serves as the basis for Cobb angle estimation. Within this framework, the SPL, Θ, and L are treated as input parameters, while the ISL, vertebral tilts, and Cobb angle constitute the outputs.

A3 Figure

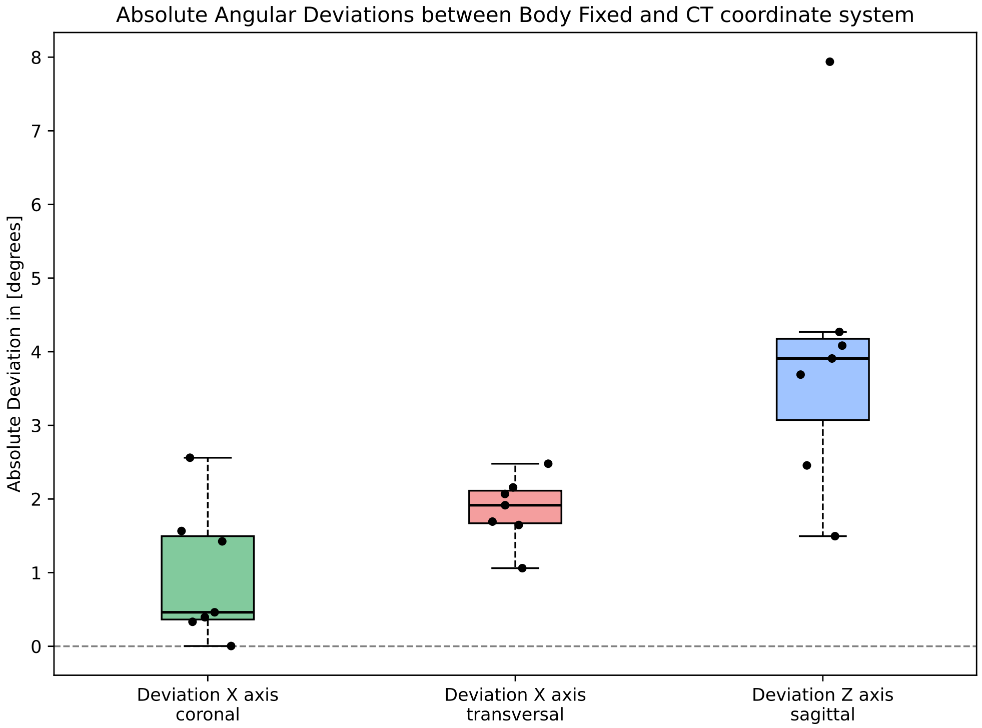

Absolute angular deviations between body fixed and CT coordinate system for the X-axis in the coronal and transversal planes, and the Z-axis in the sagittal plane.

A4 Text

1 Analysis of parameter dependencies and error propagation in Drerup's backshape-to-spine approach

This supplementary section aims to showcase the potential of using a CT extracted dataset for the validation of backshape-to-spine approaches, by providing an in depth analysis of our own implementation of the widely cited method developed by Drerup and Hierholzer [1]. In the following analysis, we utilize the systematically constructed, CT-extracted dataset to demonstrate its capability for comprehensive and rigorous analysis of parameter dependencies and error propagation in the backshape-to-spine approach developed by Drerup and Hierholzer [1]. By design, all input parameters required by this method— specifically the SPL, L, and Θ — are available both as estimates (E) and as true values (T).

This structure allows estimated outputs of Drerup's method to be generated using all possible combinations of estimated and true input parameters. For example, (E,E,E) indicates that all three parameters are estimated, while (T,E,E) denotes that only SPL is substituted with its true value. This systematic substitution enables targeted investigation of how each individual input parameter influences the final estimated output, providing detailed insight into the sources and propagation of error within the method.

We subsequently compute the Cobb angle using both the estimated and true ISL curves, employing two state-of-the-art function-fitting approaches: polynomial fitting and FMS fitting (see Sec. 1.3). Both methods are widely used in the literature, and it remains an open question as to which approach yields superior results [10–12].

We apply this verification framework to a cohort of 17 patients (Sec. 2.1 Main Manuscript), enabling examination of Drerup's method errors using this dataset across diverse spinal curvatures and three anatomical regions: complete vertebral column, curved part and straight part.

1.1 Single patient results

In this section, the outputs of our Drerup-based implementation for a randomly chosen patient are systematically illustrated across four figures. Fig 2 presents transverse anatomical slices, Fig 3 displays planar spinal trajectories, and Fig 4 depicts Cobb angle estimation. In each figure, two estimated outputs-using (E,E,E) (green) and (T,T,E) (blue) input parameter combinations-are directly compared to the true values (red). Collectively, these visualizations provide a comprehensive overview of the positional and angular estimations produced by our Drerup-based implementation relative to the true anatomical references, before input and output deviations are summarized in Fig 5(e–h).

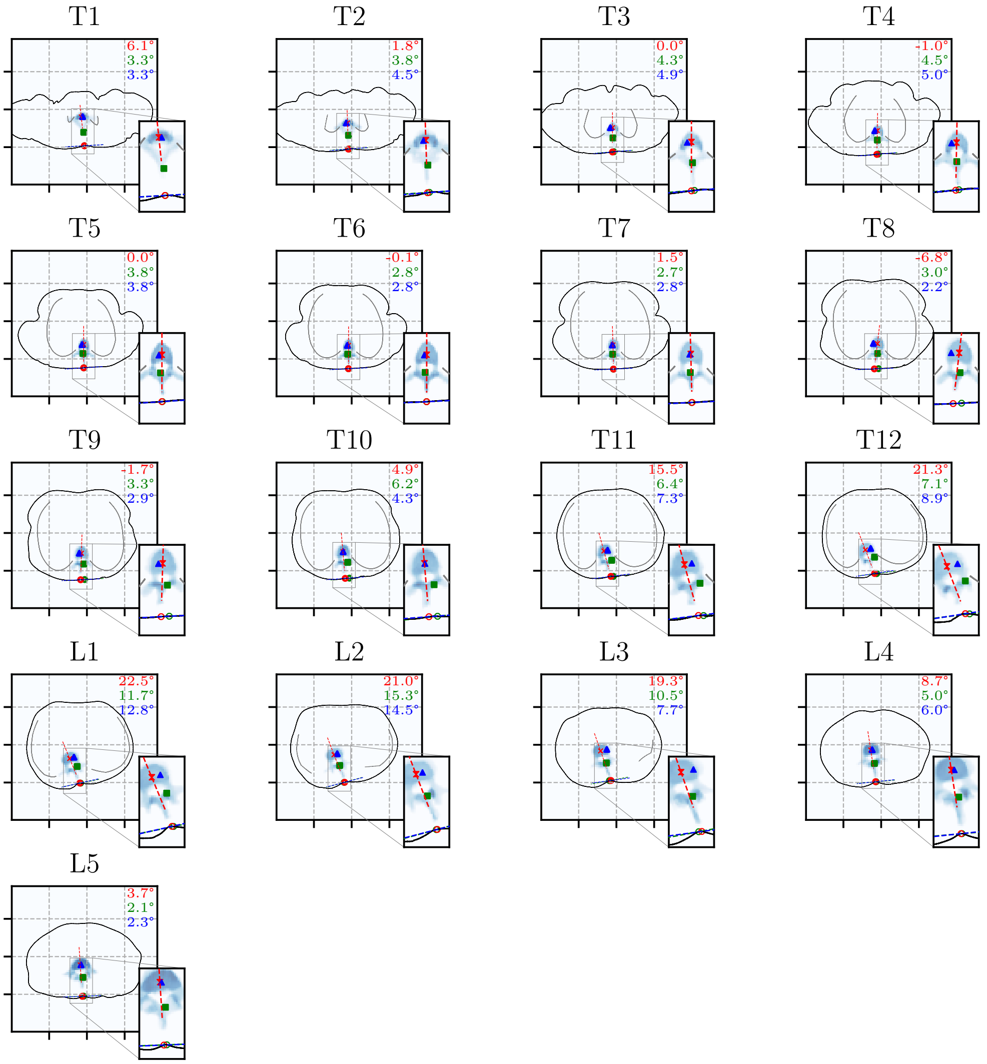

Fig 2. Transverse slices of the skin surface, ribcage surface, and vertebrae for a single patient. Markers indicate output of our Drerup-based implementation using only estimated inputs (green), ground truth values (red), and the output using true SPL and L with estimated Θ (blue). Axial vertebral rotation values for each level are shown in the top right corner of each slice in the respective colors.

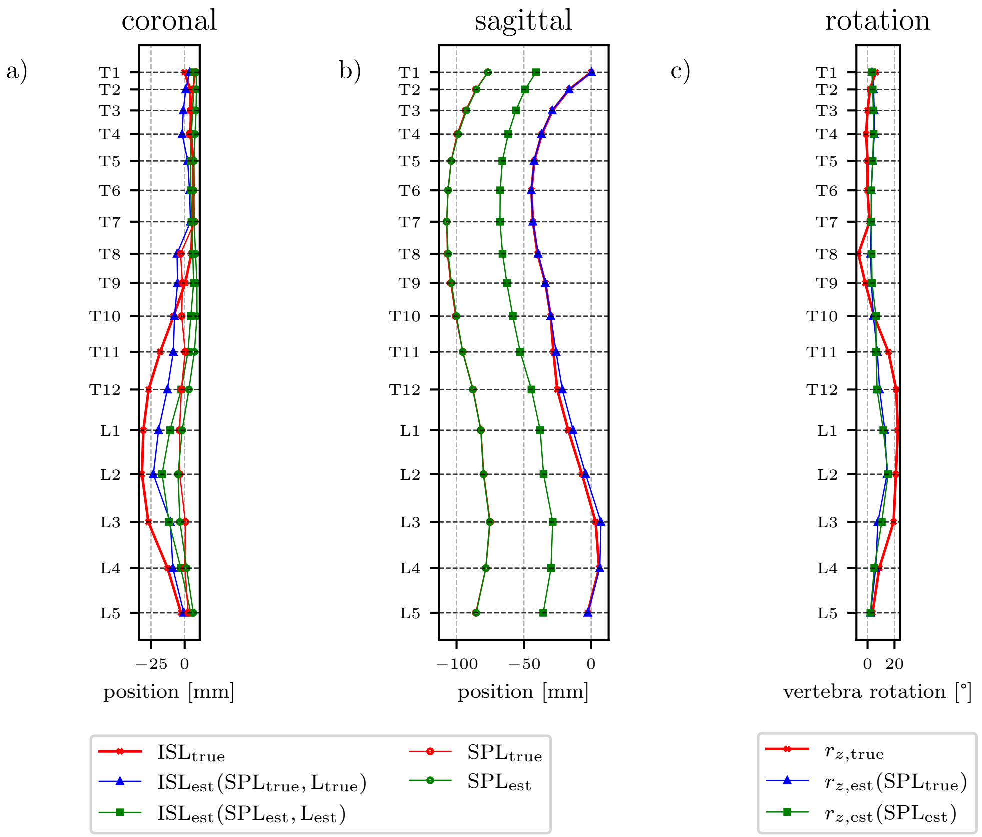

Fig 3. Comparison of our Drerup-based implementation output using estimated inputs (green) and true SPL, L, estimated Θ (blue) for a single patient (P6). (a) ISL and SPL curves in the coronal plane, (b) ISL and SPL curves in the sagittal plane, and (c) axial vertebral rotation.

a)

b)

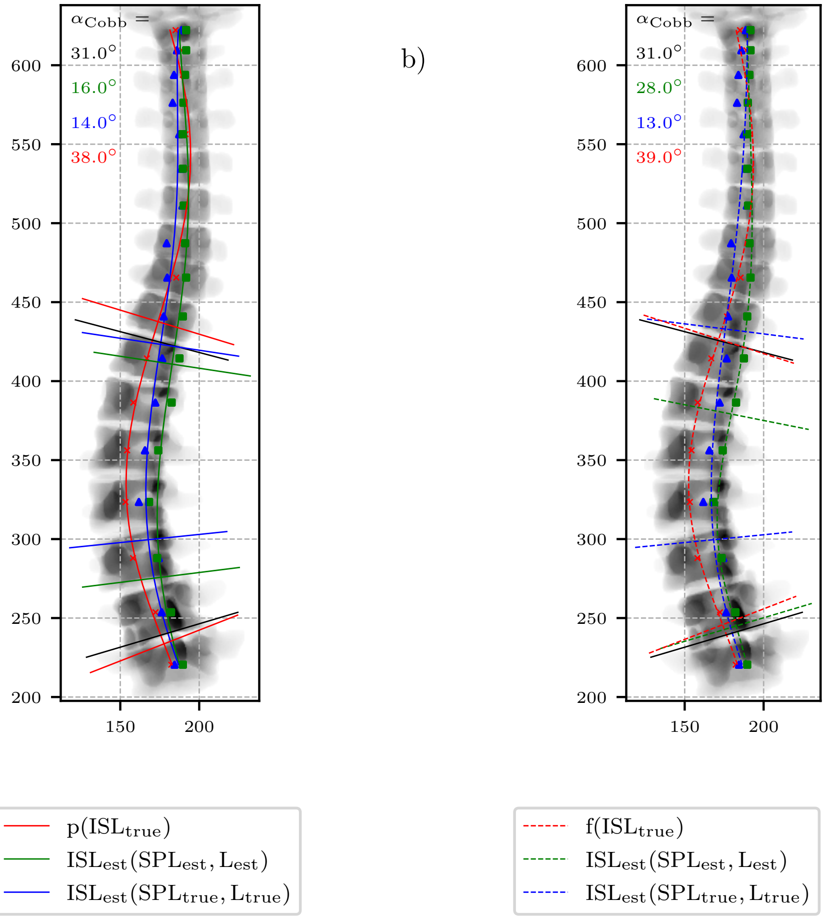

Fig 4. Cobb angle calculation from spinal trajectories using function fitting a) polynomial fit and b) frequency-modulated sine fit.

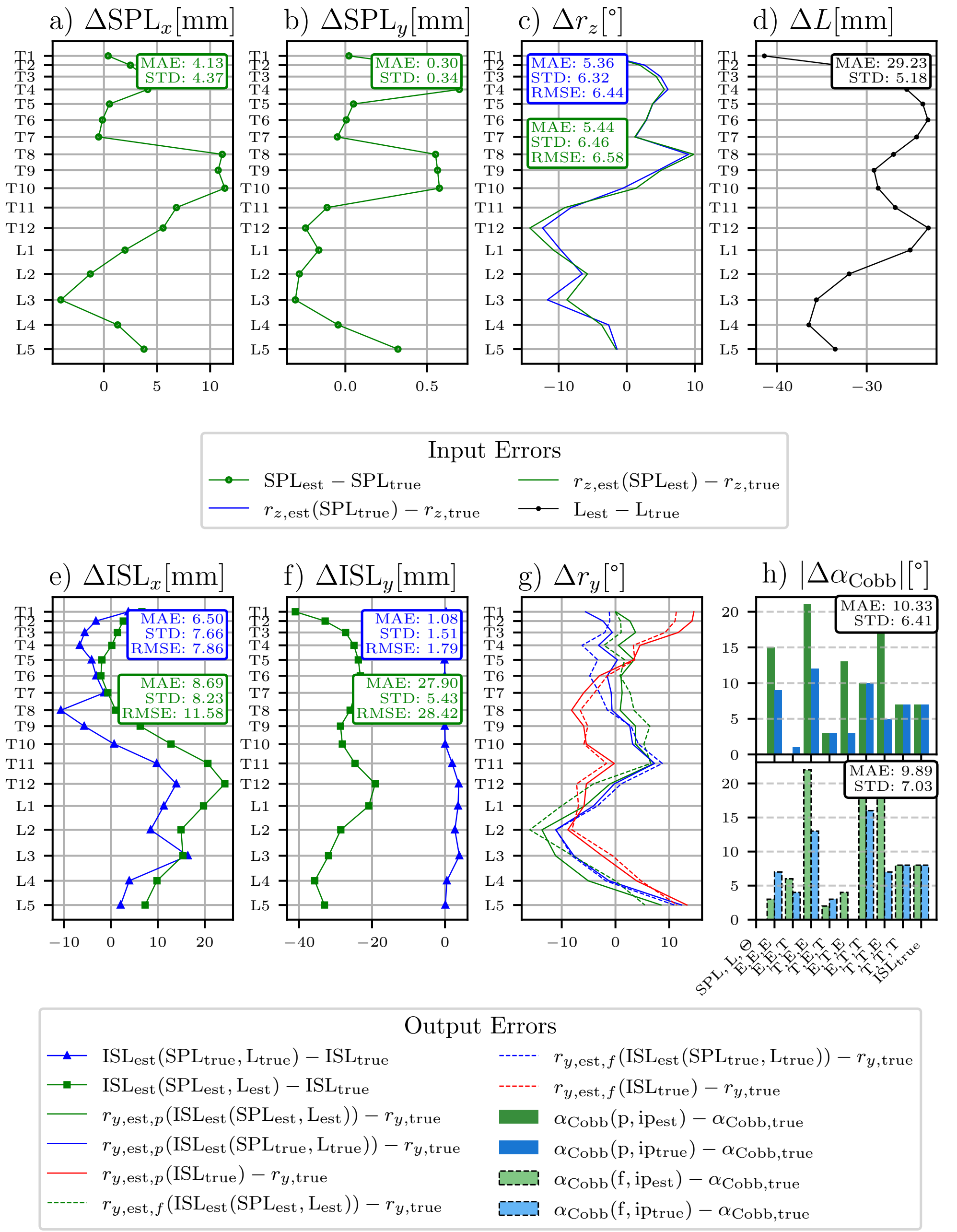

Fig 5. Deviations between our Drerup-based implementation estimated and true values for a single patient. (a) SPL deviations in the coronal plane, (b) SPL deviations in the sagittal plane, (c) axial rotation deviations, (d) distance L deviations, (e) ISL deviations in the coronal plane, (f) ISL deviations in the sagittal plane, (g) vertebral tilt deviations in the coronal plane, and (h) Cobb angle deviations. Green curves show estimates using all estimated inputs, red curves show estimates of our Drerup-based implementation applied to true values, and blue curves show estimates using true SPL and L but estimated Θ. The Cobb angle bar chart (h) is split horizontally for polynomial fit (top) and FMS fit (bottom), with results for all input parameter combinations. Mean absolute error (MAE), standard deviation (STD), and root mean square error (RMSE) are reported along vertebral levels T_1 to L_5; for (h), MAE and STD are shown across patients.

Fig 2 displays transverse anatomical slices at true vertebral centroid levels from T_1 to L_5, showing the skin surface, ribcage contour, and vertebrae. our Drerup-based implementation outputs using (E,E,E) (green) are represented as follows: squares for $ISL_{\text{est}}(z)$, rings for $\text{SPL}_{\text{est}}(z)$, and lines on the backsurface for $\Theta_{\text{SPL}_{\text{est}}(z)}$. Ground truth references (red) are shown as crosses for $ISL_{\text{true}}(z)$, rings for $\text{SPL}_{\text{true}}(z)$, and lines on the vertebra for $r_{z_c,\text{true},z}$. Outputs using (T,T,E) (blue) is indicated by triangles for $ISL_{\text{est}}(\text{SPL}_{\text{true}}(z), \text{L}_{\text{true}}(z), \Theta_{\text{SPL}_{\text{true}}(z)}, z)$. Estimated and true rotations are numerically displayed, in their respective colors, in the top-right corner of each slice. Across all vertebral levels, outputs using (E,E,E) consistently predict centroids closer to the back surface compared to (T,T,E). Deviations between estimated and true rotations are observed in most slices, across both low- and high-rotation cases. In some cases, such as at vertebral levels T_4, T_6, T_8 and T_9, the back surface is rotated in the opposite direction to the true vertebral rotation, this is also reflected in the sign difference between estimated angles and true angles. In all cases, estimates, regardless of input combination, remain within the true vertebral slice. However, centroids predicted using (E,E,E) inputs do not intersect the vertebral body, while centroids predicted using (T,T,E) consistently fall within vertebral body boundaries.

Fig 3 extends the slicewise analysis to a vertical plane view of the entire spine. The ISL and SPL curves are presented in the coronal and sagittal planes (a and b), and the axial vertebrae rotation curve is shown in (c), using consistent color coding. Both outputs, using estimated and true inputs, underestimate the ISL bending in the curved region of the coronal plane (a). The estimated and true SPL curves (red-ringed line for true, green-ringed line for estimated) are closely aligned in both the coronal (a) and sagittal (b) planes, with nearly complete overlap in the sagittal plane. In subplot (b), the estimated ISL using (E,E,E) (green squared line) is systematically positioned closer to the skin surface (near the SPL) and does not replicate the anatomical shape accurately. In contrast, the estimate using (T,T,E) (blue triangle line) closely overlaps the true ISL (red star line).

Fig 4 visualizes the Cobb angle prediction component of our Drerup-based implementation (Sec. 1.3), with all results overlaid on a 2D CT spine image for anatomical reference. Subfigures (a) and (b) display identical data points — red crosses for true values, green squares for output using (E,E,E), and blue triangles for output using (T,T,E) — but differ in the fitting method: (a) uses polynomial fitting, while (b) uses FMS fitting. Measurement points for Cobb angle determination are shown as angled lines in the color corresponding to each fit. Black lines indicate the original Cobb angle definition [13], measured between the inferior endplate of L_4 and the superior endplate of T_{11}. Numerical Cobb angle values are displayed in the top left corners in their respective colors. In both (a) and (b), the estimated Cobb angle is measured within the region where the true Cobb angle is measured (T_{11}–L_4), but does not correspond to the exact vertebral endplates used for the true Cobb angle measurement. Furthermore, the estimated curves underlying this measurement do not closely match the true spinal curve, although they are anatomically aligned with the vertebrae in the 2D flattened CT image. In both subfigures, the estimated Cobb angle based on the true ISL (red) is measured over a region similar to the true measurement (black), but the resulting angle differs from the true Cobb angle.

Subfigures (a–d) in Fig 5 quantify input parameter errors by plotting differences between estimated and true values (estimated − true) across vertebral levels T_1 to L_5. The SPL demonstrates a MAE of 4.13 mm with a STD of 4.37 mm, including maximum deviations of up to 10 mm in the coronal plane (a). Notably, these maximum deviations occur outside the region of greatest curvature (lumbar region (L_1 to L_4)), but in the transition into the straight region (T_{10} and above). In the sagittal plane (b), SPL deviations are much lower, remaining below 1 mm. The angle Θ, serving as an axial

rotation estimate ($r_{z_c,\text{est}}$) (valid under the previous assumption $K = 1$), is shown in (c). Two lines correspond to the calculation of Θ at the estimated SPL (green line) and at the true SPL (blue line). The differences between the two estimations are marginal, as evidenced by their similar RMSE (6.44°)/(6.58°) , MAE (5.36°)/(5.44°) and STD (6.44°)/(6.46°) values. The maximum deviations of Θ values are approximately 10°. Notably, axial rotation is systematically underestimated in the curved region (T_{11}–L_5) while being overestimated in large parts of the non-curved region (T_3–T_{10}). The distance L in panel (d) demonstrates a systematic underestimation (MAE = 29.23 mm) reaching maximum amplitudes of 42 mm (notably at T_1) coupled with non-systematic variability along all vertebral levels, characterized by a STD of 5.18 mm.

Subfigures (e–g) in Fig 5 summarize the deviations of output parameters (estimated − true) along vertebral levels T_1 to L_5. The Cobb angle deviations (subfigure h) are analyzed for two cases: polynomial fitting (top) and FMS fitting (bottom). The results are shown for both estimated positions (green) and true positions (blue) across input parameter combinations (E: estimated, T: true for SPL, L, and Θ). In the coronal plane (e), the deviations of ISL are larger for estimated input parameters (RSME = 11.58 mm, MAE = 8.69 mm, STD =8.23 mm, maximum = 22 mm), for true input parameters (RMSE = 7.86 mm, MAE = 6.50 mm, STD = 7.66 mm, maximum = 15 mm). In the sagittal plane (f), errors are minimal for true L (RMSE = 1.79 mm, MAE = 1.08 mm, STD = 1.51 mm) but escalate for estimated L (RMSE = 28.42 mm, MAE = 27.90 mm, STD = 5.43 mm). Moreover, the form of the curve in subfigure (f) replicates both the magnitude and spatial distribution of the deviation curve in subfigure (d). Notably, the largest deviations with true inputs are found in the curved region (T_{12}–L_5), where vertebrae exhibit the greatest rotation and our Drerup-based implementation underestimates this rotation by 8° to 12°, as shown in subfigure (c). Subfigure (g) compares vertebral tilt deviations in the coronal plane for three ISL curves: estimated inputs (green), true inputs (blue), and ground truth (red). Both polynomial (solid line) and FMS (dashed line) fits deviate markedly from the true vertebral tilts in the straight spinal region (T_1–T_{11}), failing to replicate either the numerical values or the anatomical trajectory. In the curved region (T_{12}–L_5), while numerical alignment remains imperfect, the estimated curves follow the general trajectory of the true ISL, matching its anatomical course despite positional offsets. Notably, the predicted curves (all except red) accurately reflect the true spinal inclination in the Cobb angle measurement region (T_{11}–L_4), with errors approaching zero. In contrast, the true ISL curves (red) exhibit inclination deviations of approximately 5° within these vertebral segments.

Subfigure (h) of Fig 5 demonstrates high variability in Cobb angle estimates across input parameter combinations and measurement positions, with deviations ranging from 0° to 22°. The polynomial fitting method exhibits a STD of 6.41°, while the FMS fit shows greater variability (STD = 7.03°). Within the method both FMS and polynomial fit produce identical Cobb angle estimates for the true ISL (ISL_{true}) and the outputs when using only true inputs (T,T,T), although the actual estimates differ between the two fitting methods. Notably, both fits achieve accurate Cobb angle estimations (0° error), FMS fit for (E,T,E) and polynomial fit for (E,E,T). Across all combinations, the deviations between methods also vary but despite this interchange, both methods yield comparable MAEs (MAE 10.33° polynomial vs. 9.89° FMS).

1.2 Multi patient Analysis

Section 1.1 demonstrated the calculated parameters through a detailed single-patient analysis. Building on this foundation, we now expand the evaluation to a cohort of 17 patients (P1–P17) exhibiting diverse spinal curvature types and severities. This broader analysis demonstrates how using synchronized skin surface and vertebra poses extracted from CT images enables comprehensive assessment of backshape-to-spine methods

across a wide range of spinal curvatures and anatomical regions.

Errors of input parameters are presented as bar charts in Figs 6 — 8. The y-axis indicates MAE for Figs 6 and 8 , and RMSE for Fig 7. The x-axis lists patients in a consistent order, with different parameter combinations distinguished by color. The overall mean MAE or RMSE across all patients is shown as a dashed line and as text above the bars.

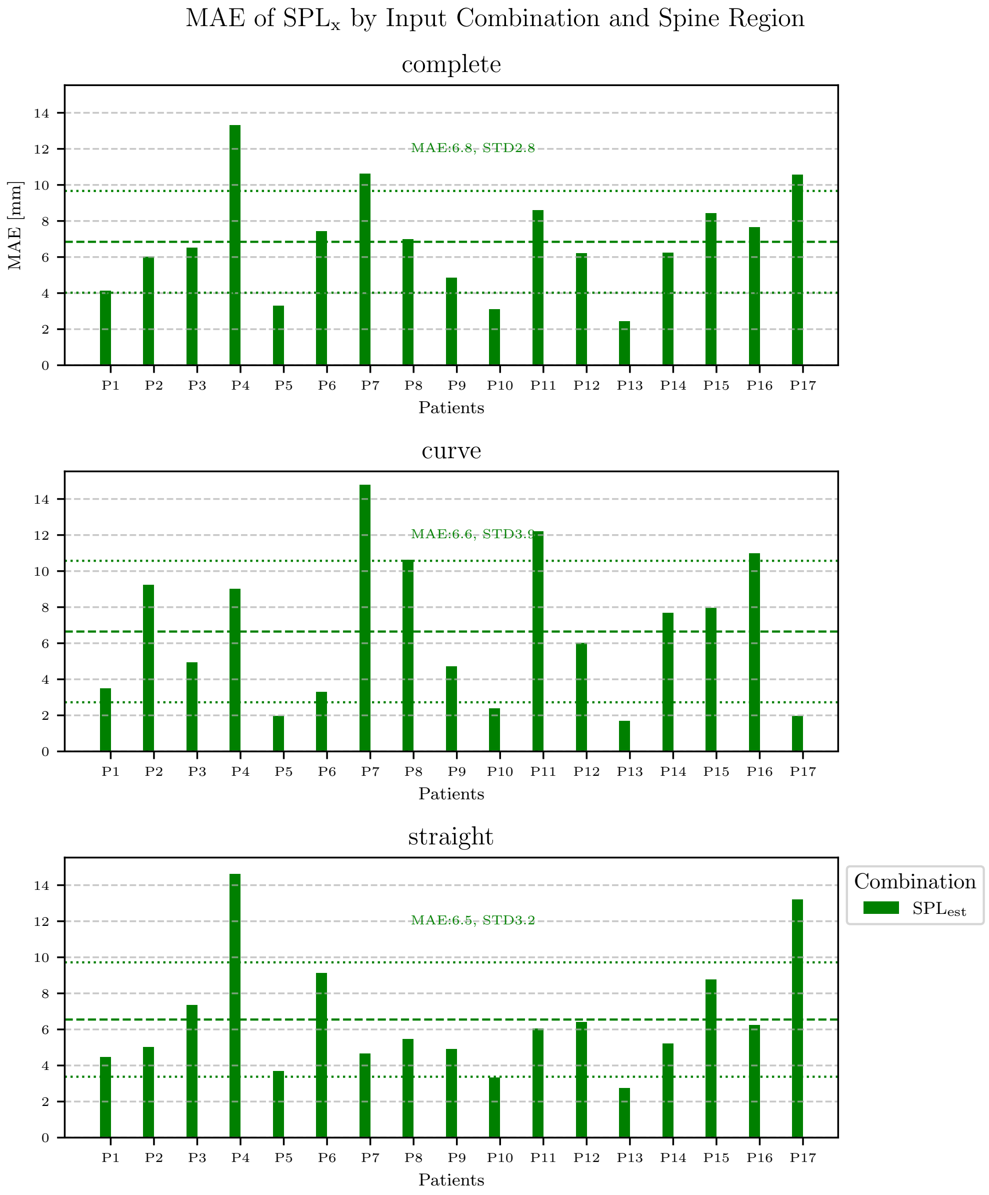

Fig 6. MAE of SPL_x evaluated from vertebral levels T_1 to L_5 for each patient represented as bar. The dashed and dotted lines represent average MAE and STD across all patients with the corresponding values above the bars.

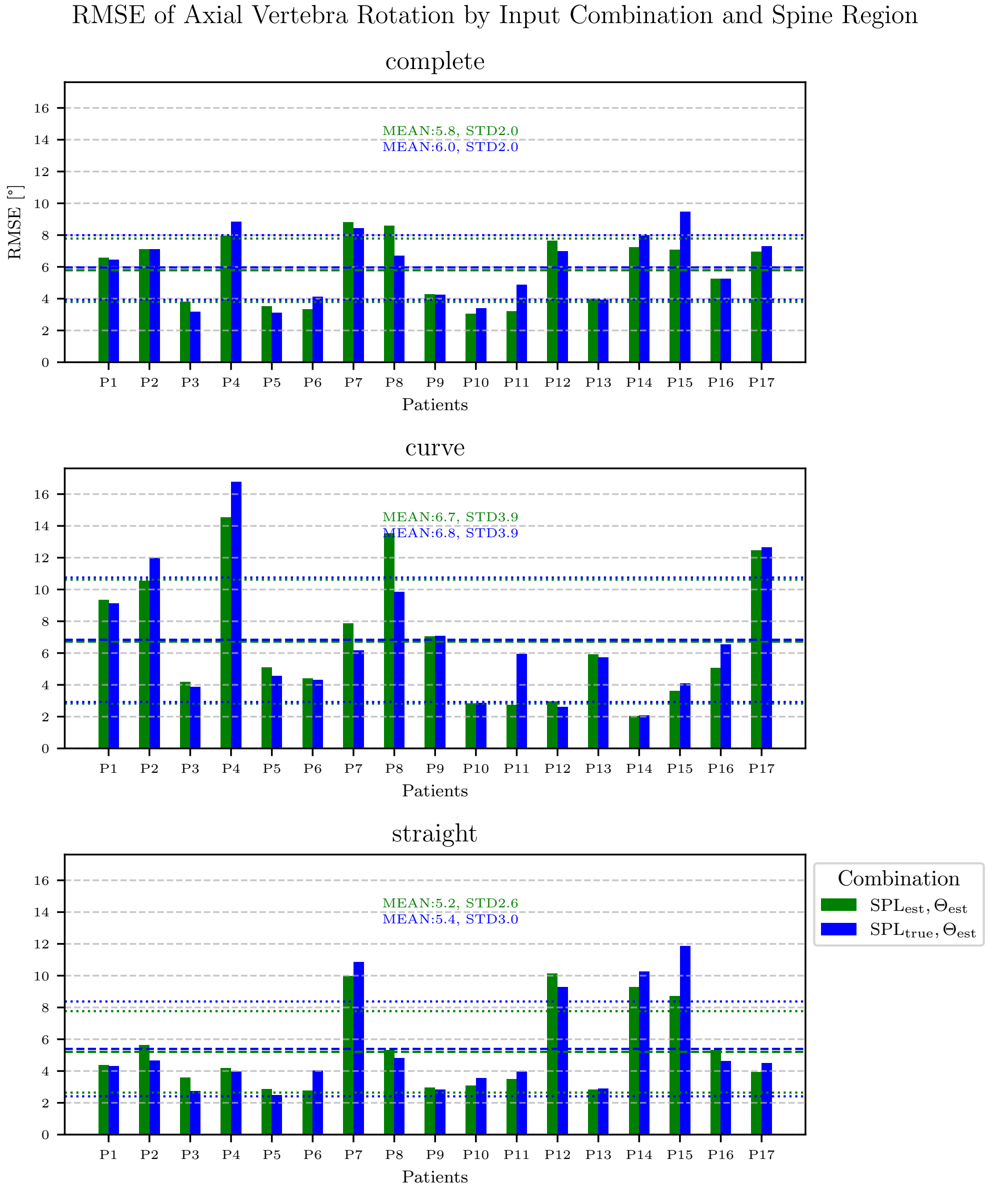

Fig 7. RMSE of axial vertebra rotation r_z evaluated from vertebral levels T_1 to L_5 for each patient represented as bar. Green bars represent r_z calculated using estimated SPL, blue bars represent r_z calculated using true SPL. The dashed and dotted lines represent average MAE and STD across all patients with the corresponding values above the bars.

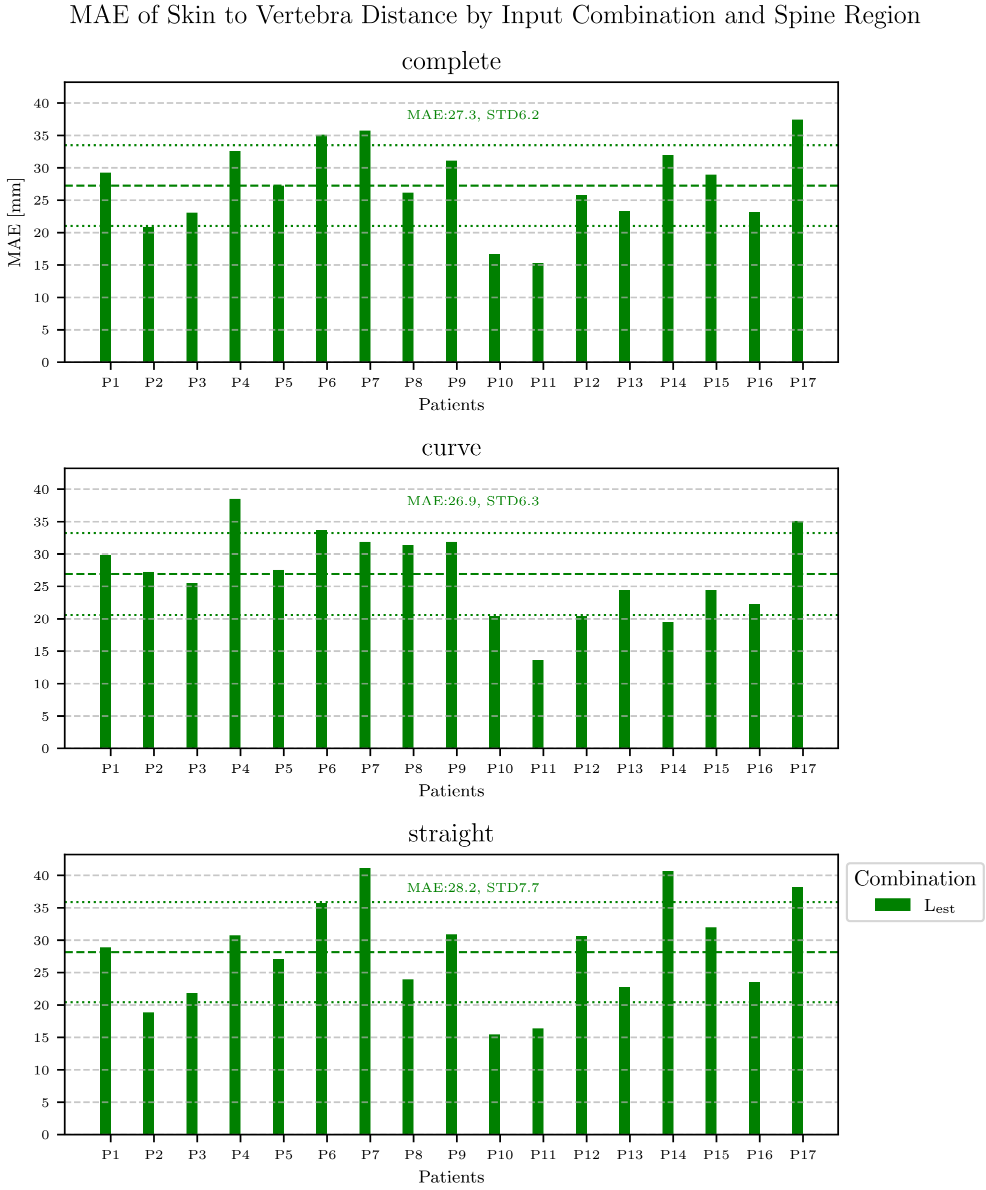

Fig 8. MAE of L evaluated from vertebral levels T_1 to L_5 for each patient represented as bar. The dashed and dotted lines represent average MAE and STD across all patients with the corresponding values above the bars.

Output errors are visualized as heatmaps: Fig 9 displays RMSE values of vertebrae centroid positions, while Fig 10 shows absolute deviations of Cobb angle. In both heatmaps, the x-axis represents individual patients sorted by curvature severity (mild to strong), and the y-axis corresponds to the input parameter combinations. The RMSE or MAE in each heatmap cell represents the error calculated along the spine at each true vertebral position from T_1 to L_5. Results are further presented for anatomical region, with separate analyses for the full spine, the curved region, and the straight region. Average MAE and STD values for each row and column in the heatmaps are presented at the ends of the corresponding rows and columns. Fig 10 further differentiates the absolute deviations in Cobb angle by patient and parameter combination, comparing

polynomial (p) and FMS (f) fitting methods, as well as estimated $\mathrm{ip}_{\mathrm{est}}$ identified by our Drerup-based implementation and true measurement $\mathrm{ip}_{\mathrm{true}}$ positions. The criterion for interpretability, defined as apex deviations exceeding one vertebral level from the anatomical reference established via the standard radiographic Cobb method, is indicated by red crosses wherever the Cobb angle is considered non-interpretable. The FMS fit yields 93 interpretable Cobb angle estimates out of 153 possible combinations, which is slightly more, compared to the polynomial fit (83 out of 153). In order to demonstrate how the method is usually applied, all results for the parameter combination (E,E,E) presented in the heatmaps and bar charts are summarized in Table 2.

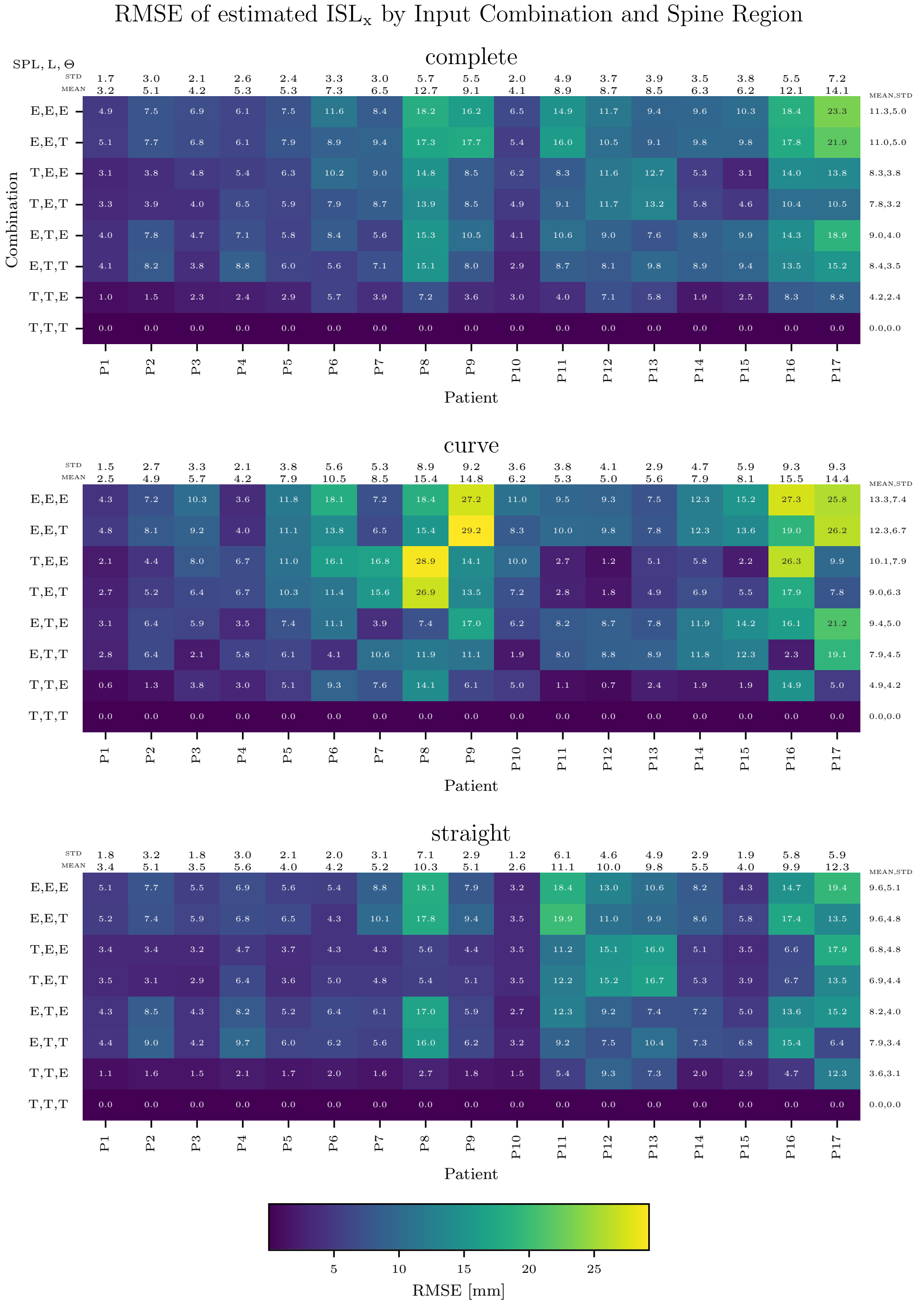

Fig 9. RMSE of vertebrae centroid positions evaluated from vertebral levels T_1 to L_5 for each patient (x-axis) and each input parameter combination (y-axis) represented as heatmap. Classification of scoliosis severity and curve type is indicated by colored frames. Average RMSE and STD across all patients and input parameter combinations are shown at the end of each row and column respectively.

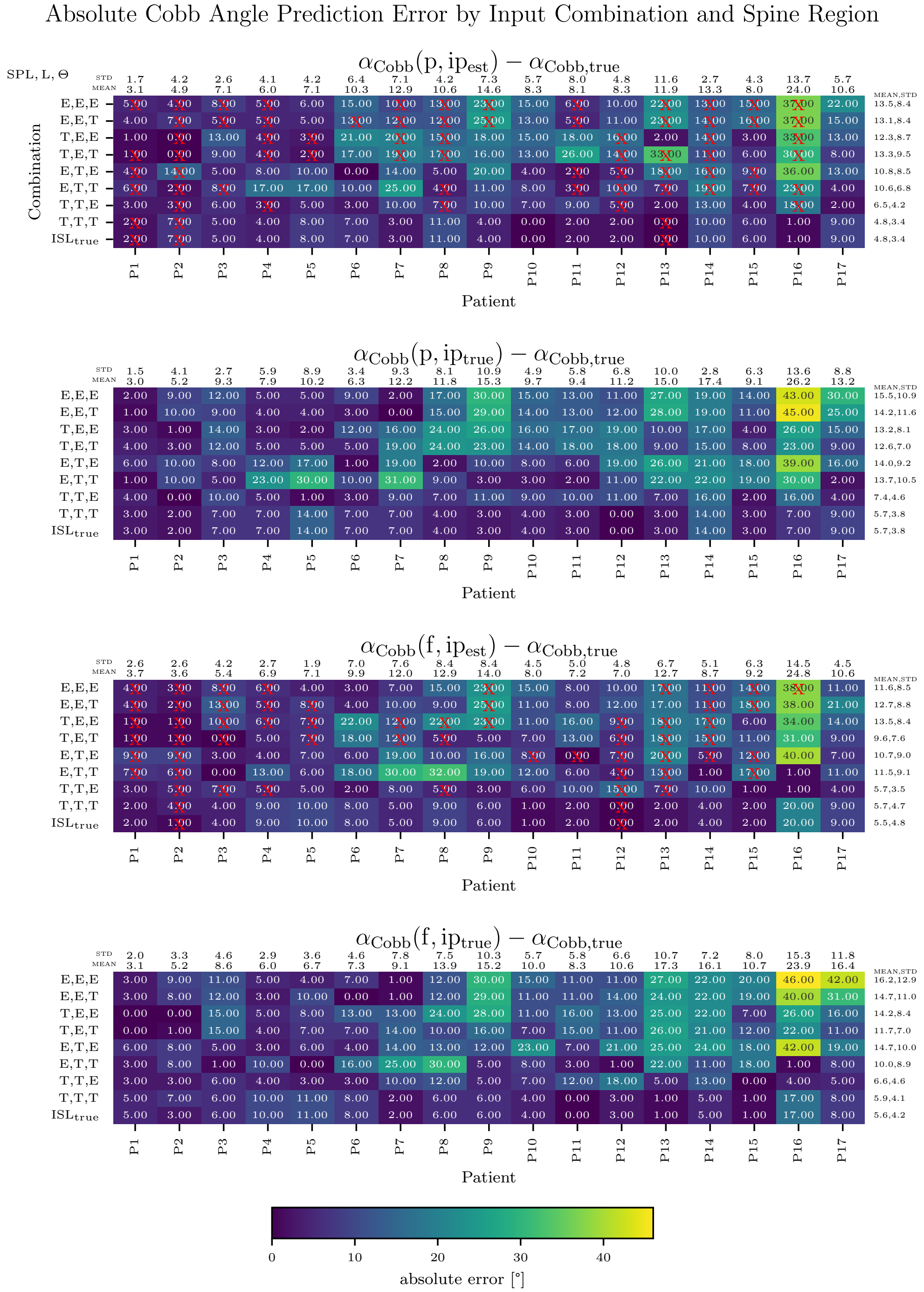

Fig 10. Heatmap visualization of absolute Cobb angle deviations across patients (x-axis) and input parameter combinations (y-axis). The top two rows displays results for polynomial fitting, while the two bottom rows corresponds to FMS fitting. Subplots one and three show deviations calculated using estimated inflection points (ip_{est}), and subplots two and 4 use ground-truth inflection points (ip_{true}). Color intensity represents the magnitude of deviations, with average mean absolute error and standard deviation displayed at the ends of each row and column. Red crosses indicate non-interpretable Cobb angle estimates, defined as deviations exceeding one vertebral level from the anatomical reference established via the standard radiographic Cobb method.

Ground truth	Cobb angle (MAE, STD)	Spine region	Vertebra rotation (mean(RMSE),STD)	ISL (mean(RMSE), STD)	SPL_x (MAE, STD)	L (MAE, STD)
CT	p:(13.5, 8.4)° f:(11.6, 8.5)°	complete	(5.8, 2.0)°	(11.3, 5.0) mm	(6.8, 2.8) mm	(27.3, 6.2) mm
		curve	(6.7, 3.9)°	(13.3, 7.4) mm	(6.6, 3.9) mm	(26.9, 6.3) mm
		straight	(5.2, 2.6)°	(9.6, 5.1) mm	(6.5, 3.2) mm	(28.2, 7.7) mm

Table 2. Summary of Drerup's [1] back-shape to spine analysis method using the CT-extracted dataset. The categories "complete," "curve," and "straight" correspond to the full spine, the curved region, and the straight region, respectively. Abbriveation p and f denote the polynomial and FMS fitting methods, respectively.

1.3 Methodological discrepancies

An examination of axial rotation estimation along the spine (Fig 5(c)) reveals distinct regional patterns. In the curved region (T_{11}–L_5), there is a significant underestimation of rotation by up to 10°, although the direction of rotation was always correctly maintained. Conversely, in the non-curved region (T_3–T_{10}), the estimated rotation direction is inverted relative to the true vertebral rotation. Importantly, these discrepancies do not reflect a limitation of the algorithm itself, but it reflects the real picture. As illustrated in Fig 2, the skin rotation in the non-curved region is indeed oriented opposite to the vertebral rotation. These findings underscore an inherent methodological constraint of surface topography: the accuracy of rotation estimation is limited by physiological factors. Body fat and musculature can obscure the relationship between the skin surface and the underlying spine. Thus the correlation between trunk deformity and radiographic spinal curvature is not always direct. Visible trunk rotation may not correspond to radiographic evidence of scoliosis, and vice versa [14, 15]. This case demonstrates that STR alone is insufficient for reliable indication of vertebral axial rotation. Moreover, applying a constant correction factor to STR, as it is described in the literature [1–3] , would only improve estimation in one region while worsening it in another, making such adjustments ineffective overall.

Another important observation concerns the relationship between deviations in surface parameters. The analysis confirms that deviations in SPL_x have minimal influence on both SPL_y and $r_{z_c,\mathrm{est}}$. Errors in SPL_y remain consistently below 1 mm, due to the skin surface's minimal y-directional variation along the x-axis (Fig 2). Similarly, the MAE and STD of $r_{z_c,\mathrm{est}}$ vary by less than 0.01 mm, regardless of whether axial rotations are estimated along the true or estimated SPL (Fig 5). This suggests that axial rotation estimations and the sagittal position of the SPL remain robust even when the SPL in the coronal plane is subject to error.

A critical methodological limitation in Cobb angle estimation from ISL is evident in the detailed analysis of results using true ISL curves (combination ISL_{true}) shown in Fig 10. These results reveal persistent Cobb angle errors independent of the fitting method: polynomial (MAE: 4.8°, STD: 3.4°) or FMS (MAE: 5.5°, STD: 4.8°) even when the true ISL is utilized(line with ISL_{true} in the first and third subplot). Errors increase substantially when using estimated input parameters (combinations E,E,E), yielding: polynomial (MAE: 13.5°, STD: 8.4°) and FMS (MAE: 11.6°, STD: 8.5°). This issue is further underscored by Fig 4(a), which highlights a fundamental mismatch in measurement regions: Cobb's original method measures the angle between the inferior endplate of vertebra L_4 and the superior endplate of T_{11}, whereas the Drerup-based implementation calculates it between T_5 and T_{11}. Additionally, Fig 10 (first and third subplot) demonstrate that in our calculations, the Drerup-based implementation produced interpretable Cobb angle estimates — defined as measurement points deviating from true positions by no more than one vertebra — only in 83 from 153 cases with polynomial fit and in 93 from 153 cases with FMS fit. This inconsistency indicates that analytical curves fitted to the ISL cannot provide reliable calculation of accurate vertebral inclinations or positions for Cobb angle estimation. These results collectively demonstrate that the current framework, even with accurate ISL reconstruction,

remains unsuitable for robust Cobb angle estimation.

The core issue of this problem stems from the method's reliance on curve fitting, which diverges from Cobb's endplate-based definition [13]. While some studies using X-ray-derived ISL curves report acceptable errors [11, 12, 16, 17], others argue that smooth curves inherently misrepresent spinal geometry, as they fail to capture discrete vertebral tilts, leading to slope mismatches at critical vertebrae [17]— a limitation also observed in the present study. Proposed solutions include implementation of statistically averaged curve slopes to approximate endplate angles [17], or entirely abandoning curve fitting and direct application of Cobb's method, which achieves higher accuracy [18–20]. Taken together, these findings emphasize that the errors originate from its fundamentally flawed assumption that analytical curves (polynomial or FMS) fitted to the ISL accurately reflect true vertebral endplate inclinations.

1.4 Effect of input parameters on ISL and Cobb angle estimation

The analysis of estimated and true input combinations (Fig 9) showed, that using input parameters (SPL, L, r_{z_c}) with true values (combination T,T,T), the Drerup-based implementation achieved perfect ISL estimation (average RMSE: 0 mm) across all patients and regions. This confirms the validity of the underlying assumptions for ISL estimation. It is important to note that, except for the case where all true inputs are used (combination T,T,T), the Drerup-based implementation achieves the lowest average RMSE when both SPL and L are estimated accurately (combination T,T,E), regardless of the spine region or patient. Conversely, using true rotation while estimating SPL and L (combination E,E,T) does not substantially improve the average RMSE compared to the case using fully estimated inputs (combination E,E,E). In contrast, when true rotation is supplied in addition to true SPL and L (transitioning from T,T,E to T,T,T), there is a marked improvement in average RMSE across all patients and regions.

These findings indicate the importance of accurate estimation of all input parameters, since our results do not allow to identify one specific parameter that is solely responsible for improving performance. However, overall, correct estimation of SPL and L appears to be more critical than accurate rotation estimation. This observation contrasts with Drerup's original assertion [1], where the importance of L, despite being not validated, was considered relatively minor for the successful application of Drerup's method.

In our study, across all patients, RMSE values for ISL deviations are consistently higher in curved spinal regions than in straight regions. The Drerup-based implementation also achieves better ISL accuracy in patients with mild curves compared to those with more severe deformities. Although this regional and curvature-dependent error pattern for ISL has not been specifically documented in previous studies, it is consistent with reports of increased Cobb angle estimation errors in more pronounced curvatures (Table 2 Main Manuscript). Overall, these findings indicate that the performance of the Drerup-based implementation decreases as curvature severity increases. However, there are notable patient-specific exceptions. For example, high RMSE of Patients P9, P16 and P17 align with large input parameter errors. However Patient P8 also shows high ISL errors despite having relatively accurate input parameters. This suggests that factors beyond input parameter accuracy, possibly including anatomical variability or other unmeasured influences, may impact algorithmic performance. A larger patient cohort will be necessary to better identify and understand these additional factors.

Further analysis of two ISL curve fitting methods (polynomial and FMS) with different input parameter combinations revealed no consistent pattern in error or interpretability, regardless of whether true or estimated inputs were used. In some cases,

using the true ISL even resulted in worse performance than using the estimated ISL (combination T,T,E). Also, no significant difference was observed between the two fitting methods, although FMS yielded slightly more interpretable results overall. Additionally, calculating the Cobb angle at the exact measurement levels defined by Cobb's original method [13] did not improve accuracy. These findings reinforce the earlier conclusion that function fitting is unsuitable for estimating Cobb angles from ISL curves. The problem lies not just in identifying the correct measurement positions, but in the inability of fitted functions to replicate the actual vertebral tilts of the true spine. Therefore, an alternative approach is required to accurately derive vertebral inclinations $r_{y_c,\mathrm{est}}$ from estimated ISL data.

2 Conclusion

This analysis systematically evaluated Drerup's backshape-to-spine approach using synchronized skin surface and vertebra poses extracted from CT images of 17 patients, isolating the influence of individual input parameters (SPL, L, and Θ) on error propagation. The investigation yielded the following key findings:

- **Input Parameter Dependency:** Accurate Internal Spine Line (ISL) reconstruction depends more heavily on the precise estimation of the symmetry profile line (SPL) and the distance L than on axial rotation (Θ). This empirical result directly challenges the original methodological assumption that the parameter L is of minor importance.
- **Physiological Limits of Axial Rotation:** Surface topography alone is fundamentally insufficient for reliable axial vertebral rotation estimation. The method systematically underestimates rotation in curved spinal regions (up to 10°) and frequently inverts the rotation direction in straight regions, as superficial musculature and body fat obscure the underlying vertebral alignment.
- **Fundamental Flaw in Cobb Angle Estimation:** The most critical methodological limitation is the reliance on analytical curve fitting to estimate the Cobb angle. Both polynomial and frequency-modulated sine (FMS) fits fail to accurately replicate discrete vertebral endplate inclinations. Consequently, the method produces substantial angular deviations (MAE ¿ 10°) and non-interpretable measurement regions, even when supplied with ground-truth ISL data.
- **Curvature Severity Degradation:** The predictive accuracy of Drerup's method consistently decreases as the severity of the spinal deformity increases. However, isolated high errors in patients with accurate input parameters suggest that unmeasured patient-specific anatomical variations also impact algorithmic performance.

Ultimately, while the geometric assumptions of Drerup's method hold true when utilizing perfect ground-truth inputs, its reliance on smooth analytical curves renders it unsuitable for robust, clinical Cobb angle calculation. Accurate assessment necessitates abandoning continuous curve fitting in favor of alternative approaches that directly derive discrete vertebral inclinations.

References

1. Drerup B, Hierholzer E. Back shape measurement using video rasterstereography and three-dimensional reconstruction of spinal shape. Clinical Biomechanics. 1994;9:28-36. doi:10.1016/0268-0033(94)90055-8.

2. Turner-Smith AR, Harris JD, Houghton GR, Jefferson RJ. A method for analysis of back shape in scoliosis. Journal of Biomechanics. 1988;21:497-509. doi:10.1016/0021-9290(88)90242-4.

3. Stokes IAF, Armstrong JG, Moreland MS. Spinal deformity and back surface asymmetry in idiopathic scoliosis. Journal of Orthopaedic Research. 1988;6:129-37. doi:10.1002/jor.1100060117.

4. Drerup B, Hierholzer E. Automatic localization of anatomical landmarks on the back surface and construction of a body-fixed coordinate system. Journal of Biomechanics. 1987;20:961-70. doi:10.1016/0021-9290(87)90325-3.

5. of the international Society on Scoliosis Orthopaedic M, (SOSORT) RT, Kotwicki T, Negrini S, Grivas TB, Rigo M, et al. Methodology of evaluation of morphology of the spine and the trunk in idiopathic scoliosis and other spinal deformities - 6th SOSORT consensus paper. Scoliosis. 2009;4:26. doi:10.1186/1748-7161-4-26.

6. Patias P, Grivas TB, Kaspiris A, Aggouris C, Drakoutos E. A review of the trunk surface metrics used as Scoliosis and other deformities evaluation indices. Scoliosis. 2010;5:12. doi:10.1186/1748-7161-5-12.

7. Choi R, Wataname K, Fujita N, Ogura Y, Matsumoto M, Demura S, et al. Measurement of Vertebral Rotation from Moire Image for Screening of Adolescent Idiopathic Scoliosis. The Institute of Image Electronics Engineers of Japan. 2018. doi:10.11371/tievciieej.6.2_56.

8. Grünwald ATD, Roy S, Alves-Pinto A, Lampe R. Predicting spinal column profile from surface topography via 3D non-contact surface scanning. PLOS ONE. 2021;16. doi:10.1371/journal.pone.0243736.

9. Grünwald ATD, Roy S, Lampe R. Measurement of distances and locations of thoracic and lumbar vertebral bodies from CT scans in cases of spinal deformation. BMC Medical Imaging. 2024;24:109. doi:10.1186/s12880-024-01293-6.

10. Drerup B, Hierholzer E. Assessment of scoliotic deformity from back shape asymmetry using an improved mathematical model. Clinical Biomechanics. 1996;11:376-83. doi:10.1016/0268-0033(96)00025-3.

11. Safari A, Parsaei H, Zamani A, Pourabbas B. A Semi-Automatic Algorithm for Estimating Cobb Angle. Journal of Biomedical Physics and Engineering. 2019;9. doi:10.31661/jbpe.v9i3Jun.730.

12. Russo AP, Pastorello Y, Dénes L. Automated Cobb Angle Measurements for Scoliosis Diagnosis and Assessment: AI Applications and Accuracy Enhancement Through Image Processing Techniques. Cureus. 2024. doi:10.7759/cureus.66736.

13. Cobb J, Cobb JR, Cobb JR. Outlines for the study of scoliosis. Journal of Bone and Joint Surgery, American Volume. 1948;5:261-75.

14. Goldberg CJ, Kaliszer M, Moore DP, Fogarty EE, Dowling FE. Surface Topography, Cobb Angles, and Cosmetic Change in Scoliosis. Spine. 2001;26:E55-63. doi:10.1097/00007632-200102150-00005.

15. Kotwicki T, Kinel E, Chowańska J, Bodnar-Nanus A. POTSI, Hump Sum and Sum of Rotation - New surface topography parameters for evaluation of scoliotic deformity of the trunk. Fizjoterapia Polska. 2008;8:231-40.

16. Choi R, Watanabe K, Jinguji H, Fujita N, Ogura Y, Demura S, et al. CNN-based Spine and Cobb Angle Estimator Using Moire Images. The Institute of Image Electronics Engineers of Japan. 2017. doi:10.11371/tievciieej.5.2_135.

17. Li K, Gu H, Colglazier R, Lark R, Hubbard E, French R, et al. Deep learning automates Cobb angle measurement compared with multi-expert observers. arXiv. 2024. doi:10.48550/ARXIV.2403.12115.

18. Hayashi D, Regnard N, Ventre J, Marty V, Clovis L, Lim L, et al. Deep learning algorithm enables automated Cobb angle measurements with high accuracy. Skeletal Radiology. 2024. doi:10.1007/s00256-024-04853-7.

19. Li H, Qian C, Yan W, Fu D, Zheng Y, Zhang Z, et al. Use of Artificial Intelligence in Cobb Angle Measurement for Scoliosis: Retrospective Reliability and Accuracy Study of a Mobile App. Journal of Medical Internet Research. 2024;26. doi:10.2196/50631.

20. Pan Y, Chen Q, Chen T, Wang H, Zhu X, Fang Z, et al. Evaluation of a computer-aided method for measuring the Cobb angle on chest X-rays. European Spine Journal. 2019;28:3035-43. doi:10.1007/s00586-019-06115-w.
